# What Do Oral Drugs Really Look Like? Dose Regimen, Pharmacokinetics, and Safety of Recently Approved Small-Molecule Oral Drugs

**DOI:** 10.1021/acs.jmedchem.5c02863

**Published:** 2025-11-18

**Authors:** Dean G. Brown

**Affiliations:** Jnana Therapeutics, One Design Center Pl Suite 19-400, Boston, Massachusetts 02210, United States

## Abstract

An analysis of dose, dose frequency, human pharmacokinetics, and potential drug–drug interactions (DDI) was performed on small-molecule oral drugs approved by the FDA from 2020 to 2024 (*n* = 104). Although most oral drugs are administered QD (67%), BID and TID regimens are also regularly approved (32%). First-in-class (FIC) drugs and drugs with Orphan Drug Designation (ODD) have a higher frequency of BID or TID administration compared to drugs without those designations (BID and TID = 50% for FIC drugs vs 19% for non-FIC; BID and TID = 41% for ODD vs 20% non-ODD). Most drugs are >95% plasma protein bound (58%), with a large fraction >99% bound (29%). Of these drugs, 22% have black box warnings and 42% list contraindications. An examination of DDI revealed the most frequent warning around CYP3A4 induction (60%). These findings will help medicinal chemists better understand and predict typical and nontypical profiles of oral drugs.

## Graphical abstract

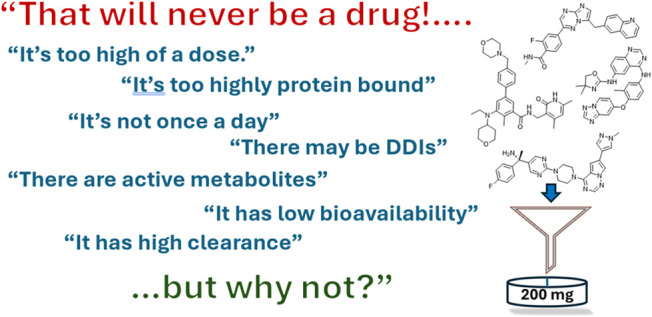

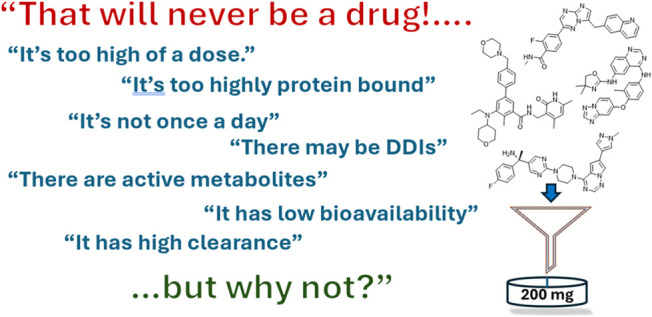

## Statement of Significance

Although it is highly desirable to aim for low dose and once daily dosing (QD) for drug discovery programs, there are important examples of recently approved drugs with high daily dosing and/or more frequent dosing regimens such as BID and even TID.

This perspective highlights the examples of approved drugs that may be considered exceptions to what makes an oral drug, such as high clearance, high plasma protein binding (PPB), low oral bioavailability, and potential for drug–drug interactions (DDI).

By highlighting the range of pharmacokinetic properties, dose regimens, and warnings in the labels of recently approved drugs, this article aims to encourage those in drug discovery to avoid being overly prescriptive when considering what profile will result in an oral drug.

## Introduction

The journey to discover and launch new drugs is a long, risky, and expensive endeavor. Most drug discovery programs fail, resulting in only a small number of compounds being approved as new drugs each year. Approximately 30–50 new drugs are approved by the FDA every year, with ∼70% of those being small molecules.^1^ In the most recent report, it was estimated that the biopharmaceutical industry as a whole invested $276 billion in research and development in 2021.^2^ For individual drug discovery and development companies, it is estimated that from start to launch, a typical program may cost the sponsor ∼$1.1 billion (accounting also for expenditures on failed programs and trials).^3^ Broken down by cost of phases, Sertkaya et al. reported the mean costs per program across phases,^4^ which is illustrated in Figure 1. It is difficult to find reliable measures of phase costs for nonclinical studies since various companies may have different definitions as to what constitutes each phase. The inset panel in Figure 1 illustrates in a qualitative way the increasing costs from Hit Generation ($) to Investigational New Drug ($$$$) enabling studies based on this author’s experience.

**Figure 1:**
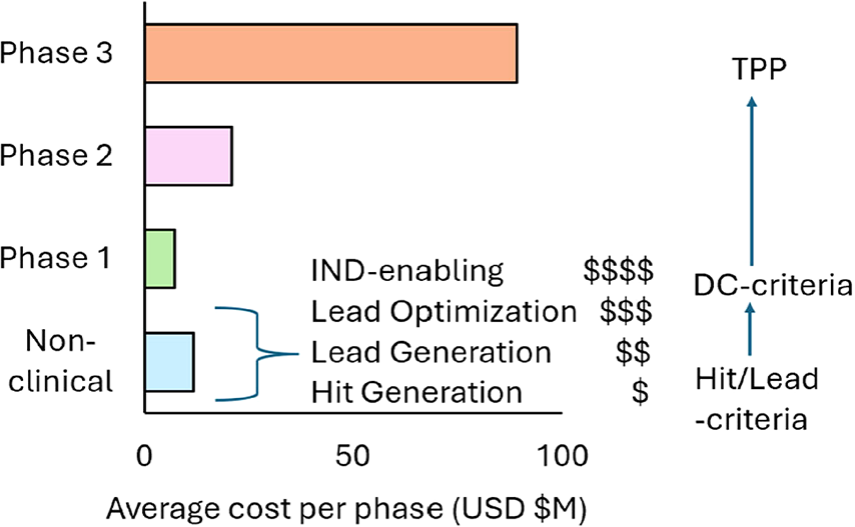
Cost per phase by average cost in USD ($M). Right hand panel is an illustration of typical criteria used in discovery and development.

One strategy the industry has adopted to help hedge their bets on this process is to “fail early” and avoid expensive Phase 3 trials, particularly those which are of high risk to deliver on a registrational end point. The term “fail” may also be translated as “win” for the company if they truly can identify an intractable risk as early as possible such as mechanism-based toxicity or invalidation of the target in discovery and thus avoid expensive late discovery or development costs (Figure 1). Furthermore, large companies may take a portfolio view of their projects and assign a probability of success of individual projects in comparison to one another and subsequently use an “attrition-based model”. The attrition-based model would prioritize those programs with the highest chance of technical, clinical, and commercial success to move forward. Those that show higher risk against those categories compared to other programs may slow down or even stop. The probability of success in these models is often measured by defining specific and generic risks to the program at the early stages based on any external or internal information that may be available. As the program moves forward, data is collected, and the team can begin to pressure-test their data against defined categories that are meant to assess drug-likeness and target validation. This data may define a key “go/no-go decision” for the team, which if positive will help to discharge a key risk to the program. If the decision is negative, the program may stop. From the biology side, such examples may include proving cellular functional activity with a project compound, establishing efficacy in an animal model, or linking target engagement to an in vivo pharmacodynamic (PD) or efficacy response. On the chemistry side, these “go/no-go” decisions are often more generic across programs. Initially, these may be defined by in vitro assays such as target potency, solubility, in vitro clearance, in vitro permeability, in vitro secondary pharmacology (such as hERG), and in silico safety assessments as examples. If these risks seem manageable, the project may move further along and define success in more complicated and expensive studies such as pharmacokinetic (PK) assessment in various species (e.g., demonstration of oral bioavailability and low in vivo clearance), preclinical toxicology studies, and ultimately Good Laboratory Practice toxicology studies. This “attrition model” relies on the assumption that early models are both available and predictive toward gauging the probability of success in clinical development. This may be true in many cases such as target validation, but in some cases, chemical risks may be hard to judge, especially in the early stages of a program. Who is to say for certain that a chemical series that has a myriad of poor drug-like properties cannot be optimized by a creative medicinal chemist given enough time and resources? As a practical matter, however, a high-risk profile for an early hit or lead series may make it more difficult to want to spend money on more expensive studies such as in vivo PK or preclinical safety. Nonetheless, the industry is full of anecdotal tales of the “chemist who wouldn’t take no for an answer” and pushed a program forward despite all of the risks and divergent opinions. One method that has been adopted by the industry to help gauge and quantify these early compound risks is the use of property-based drug design to highlight generic risks associated with a given scaffold, using mostly in silico as well as some early in vitro profiling. In this manuscript, property-based drug-design refers to the use of simple physicochemical properties or calculated physicochemical properties that are useful in predicting drug-like properties, such as logP, polar surface area, H-bond donors or acceptors (HBA or HBD), ligand efficiencies, and lipophilic efficiency as examples. Property-based drug design has been highly influential in drug discovery as one method to ascertain risks in chemical series.^5^ Many of these property-based design concepts are underpinned by an analysis of known drugs and clinical candidates. In our recent analysis of approved drugs from 2010 to 2019 for example, most oral drugs do follow Ro5 guidelines (83% with zero or one Ro5 “violation”).^1^ The use of property-based drug design using simple physicochemical predictors may be most applicable to analyzing and prioritizing screening hits or perhaps to try and correlate these properties to in vitro assays (such as hERG, microsomal clearance, etc.) as a chemical series moves from Lead Identification and into Lead Optimization. However, once a project team begins to narrow down on a lead series and select advanced compounds for in vivo experiments, what can be said about the probability of success and drug-likeness? Property-based drug design approaches in this phase of the program are now typically used to try and find relationships to develop hypotheses for addressing off-target activity (such as hERG) or poor oral absorption or brain penetration as examples. Are they predictive of in vivo DMPK? Can they predict activity in safety assays? In some cases, they may be useful for such purposes, but as the program moves deeper toward a clinical candidate, these property-based design measures are superseded with more and more complicated data, such as in vivo PK across various species, or early preclinical toxicology studies. How then is a chemist to judge how far away they are from a potential drug candidate at this point? One way that is often used to help bridge the chasm from hits to a clinical candidate may be implementation of a set of development criteria (“DC criteria” in Figure 1). These criteria may lay out in specific detail the in vivo PK properties that are required, the PD response that is required, efficacy in a model of disease, in vivo safety margins, off-target pharmacology, chemical scale up synthesis risks, intellectual property, and projected human dose as examples. The DC criteria will also align toward the Therapeutic Product Profile (TPP) that provides the long-term vision and line-of-sight into the future product label. A typical TPP will often aim to define the indication, specific patient populations, dosage, contraindications, and other properties which help to engage the sponsor with the regulatory agency around labeling concepts.^6^

One tool that helps guide a team from LO to DC criteria to the TPP is the projected human dose and exposure. The human dose needed to hit maximal efficacy with an appropriate safety margin is in essence an amalgamation of various drug-like properties such as potency, absorption, systemic exposure, clearance, and distribution. Doses which are too high or too frequent may be problematic for multiple reasons such as patient compliance, difficulty in swallowing large pills, and/or ability to formulate a tolerable pill size. In addition, several papers have linked higher daily doses to potential safety risks. For example, Jackson et al. reported in a 2022 analysis that the best models to predict drug-induced liver injury (DILI) risk were based on dose, logP, and Fsp^3,7^ An earlier paper by Aleo et al. also linked higher daily dose to higher probability of idiosyncratic drug toxicity.^8^ The term “body burden” is used to help conceptualize the idea that the parent drug and subsequent metabolites must be eliminated after a given dose. High exposures that are needed to illicit a pharmacological response may have a higher probability that the parent drug or metabolite may have a side effect or reactivity that would not be present or would be easily cleared by the body at a lower plasma concentration.

It is likely that individual organizations have specific DC criteria that are based on their internal historical case studies or recent learnings. There is no uniform set of guidelines for a DC that can be found in the literature which would translate easily across organizations. Some organizations may be more risk tolerant to specific criteria and others may not. For example, what should DC criteria be for predicted human dose, exposure, and clearance? What about the tolerance to any DDI? In order to help calibrate what drugs really look like, an analysis of dose, dose regimen, human pharmacokinetics, and potential DDI was thus performed on all small-molecule drugs approved by the FDA from 2020 to 2024 (*n* = 104). Data was extracted from labels and regulatory approval documents as accessed through the FDA Web site and is illustrated in Table 1.^9^ Table 1 summarizes new small molecule drugs approved in 2020 (22 drugs), [Bibr ref10]−[Bibr ref11] [Bibr ref12] [Bibr ref13] [Bibr ref14] [Bibr ref15] [Bibr ref16] [Bibr ref17] [Bibr ref18] [Bibr ref19] [Bibr ref20] [Bibr ref21] [Bibr ref22] [Bibr ref23] [Bibr ref24] [Bibr ref25] [Bibr ref26] [Bibr ref27] [Bibr ref28] [Bibr ref29] [Bibr ref30] [Bibr ref31] [Bibr ref32] [Bibr ref33] [Bibr ref34] [Bibr ref35] [Bibr ref36] [Bibr ref37] [Bibr ref38] [Bibr ref39] [Bibr ref40] [Bibr ref41] [Bibr ref42] [Bibr ref43] [Bibr ref44] [Bibr ref45] 2021 (24 drugs), [Bibr ref46]−[Bibr ref47] [Bibr ref48] [Bibr ref49] [Bibr ref50] [Bibr ref51] [Bibr ref52] [Bibr ref53] [Bibr ref54] [Bibr ref55] [Bibr ref56] [Bibr ref57] [Bibr ref58] [Bibr ref59] [Bibr ref60] [Bibr ref61] [Bibr ref62] [Bibr ref63] [Bibr ref64] [Bibr ref65] [Bibr ref66] [Bibr ref67] [Bibr ref68] [Bibr ref69] [Bibr ref70] [Bibr ref71] [Bibr ref72] [Bibr ref73] [Bibr ref74] [Bibr ref75] [Bibr ref76] [Bibr ref77] [Bibr ref78] 2022 (12 drugs), [Bibr ref79]−[Bibr ref80] [Bibr ref81] [Bibr ref82] [Bibr ref83] [Bibr ref84] [Bibr ref85] [Bibr ref86] [Bibr ref87] [Bibr ref88] [Bibr ref89] [Bibr ref90] [Bibr ref91] [Bibr ref92] [Bibr ref93] [Bibr ref94] [Bibr ref95] [Bibr ref96] [Bibr ref97] [Bibr ref98] [Bibr ref99] [Bibr ref100] 2023 (25 drugs),^101–133^ and 2024 (21 drugs).^134–162^

**Table 1:**
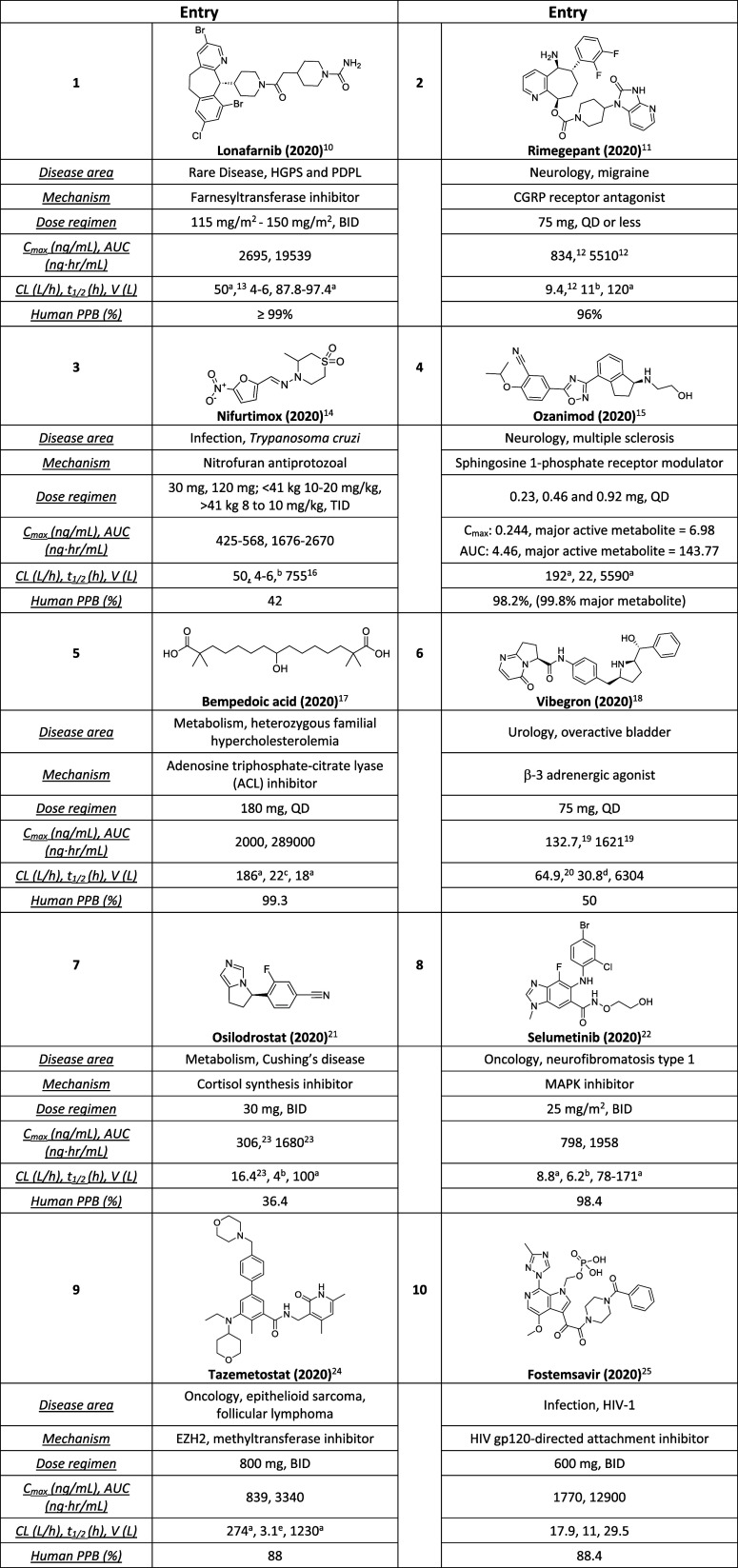

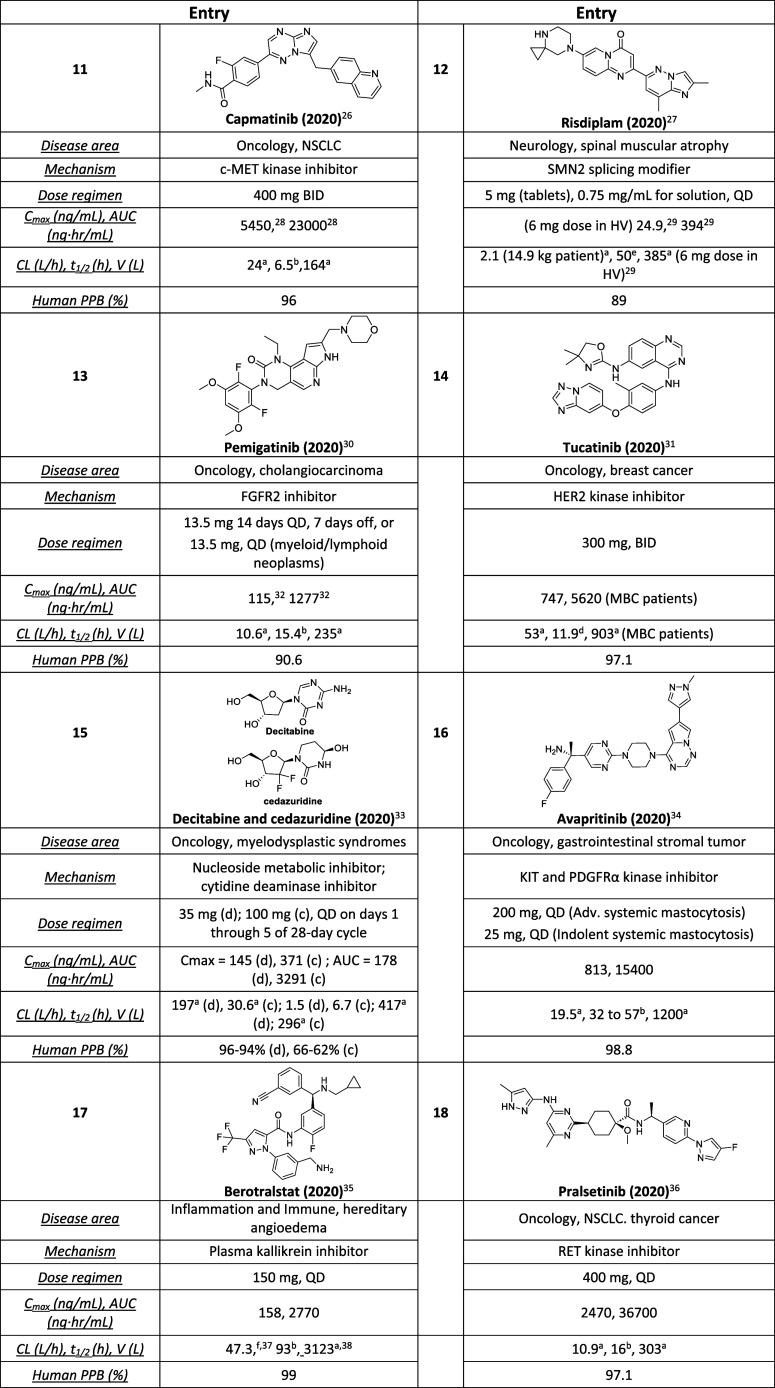

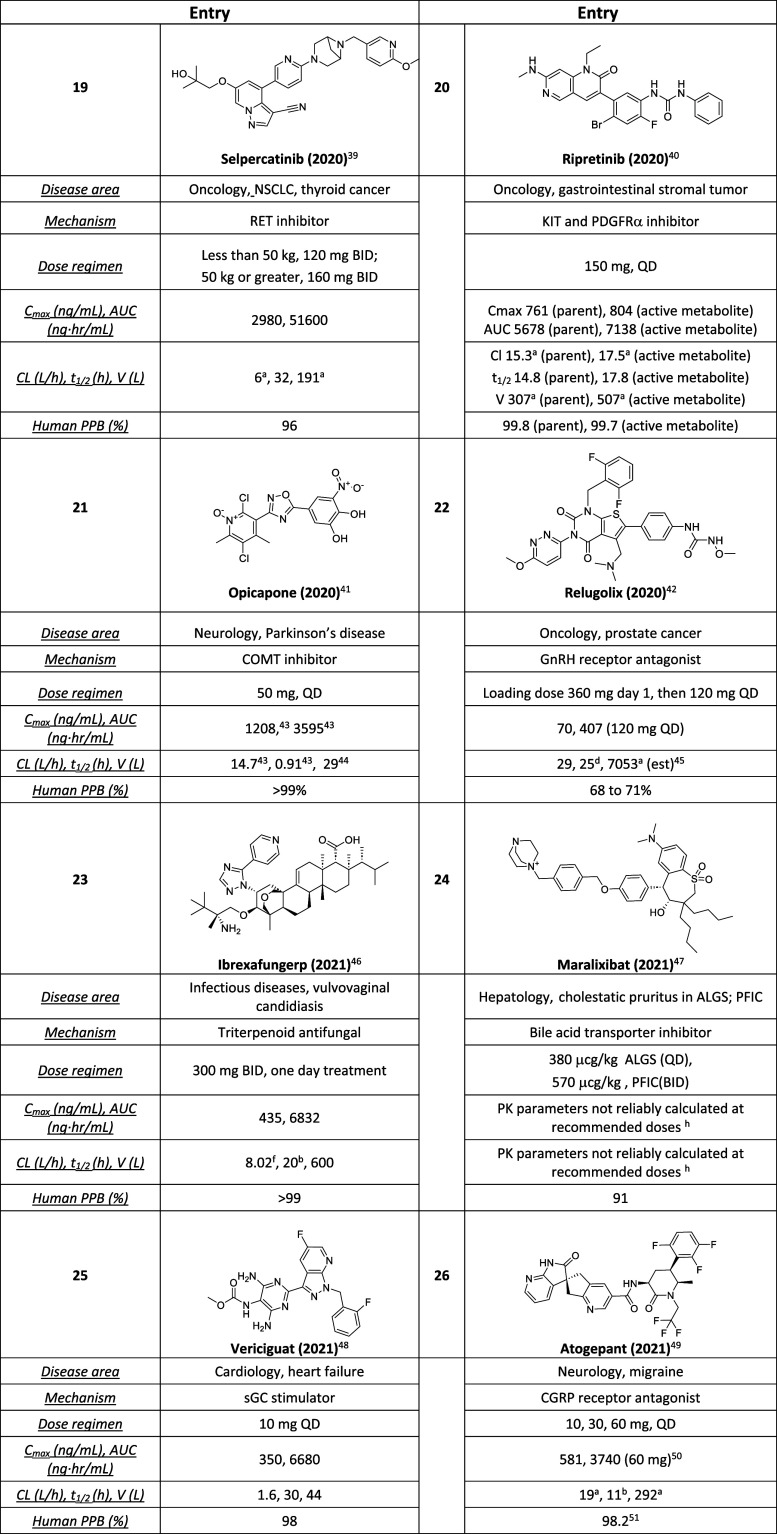

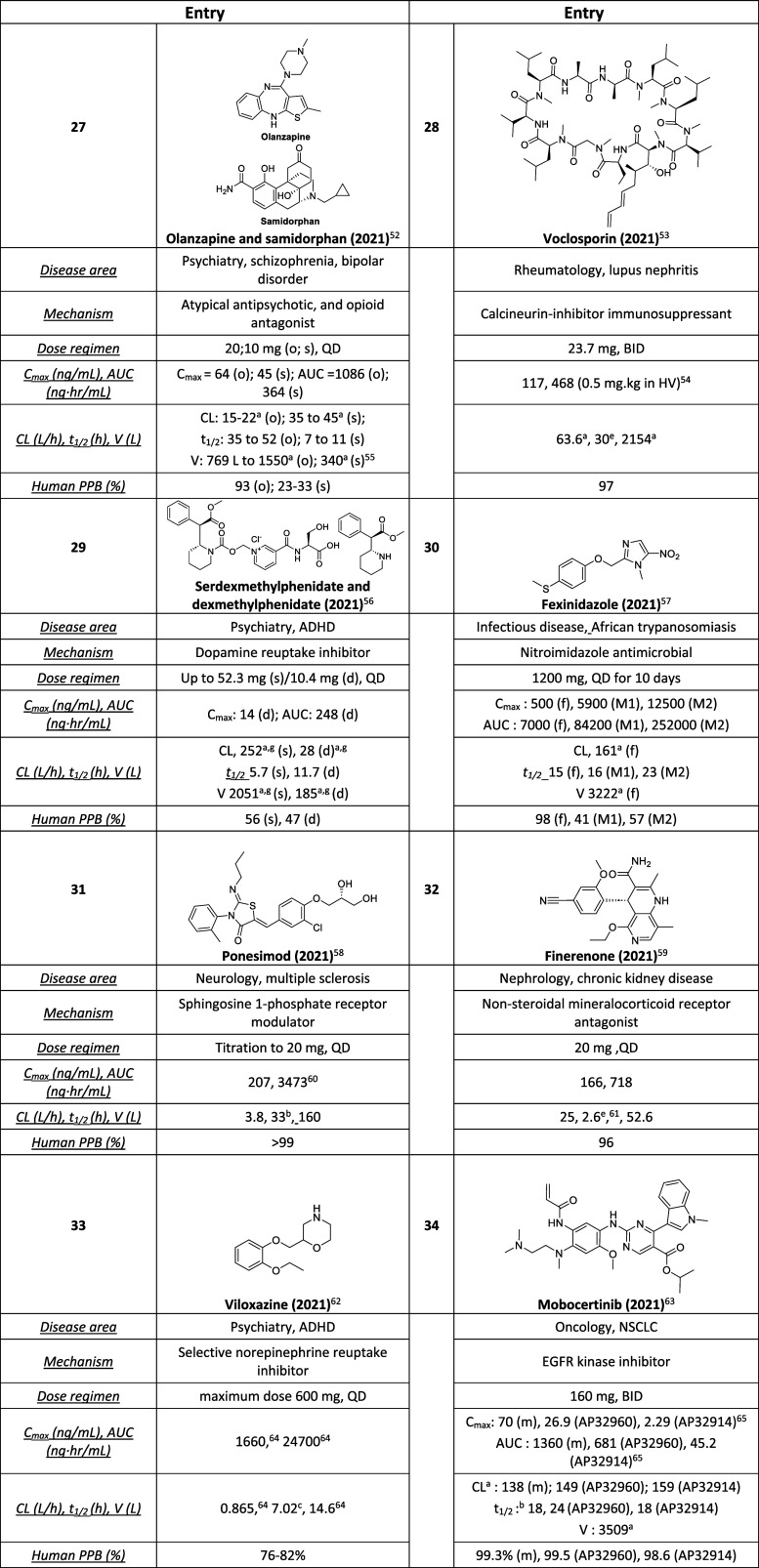

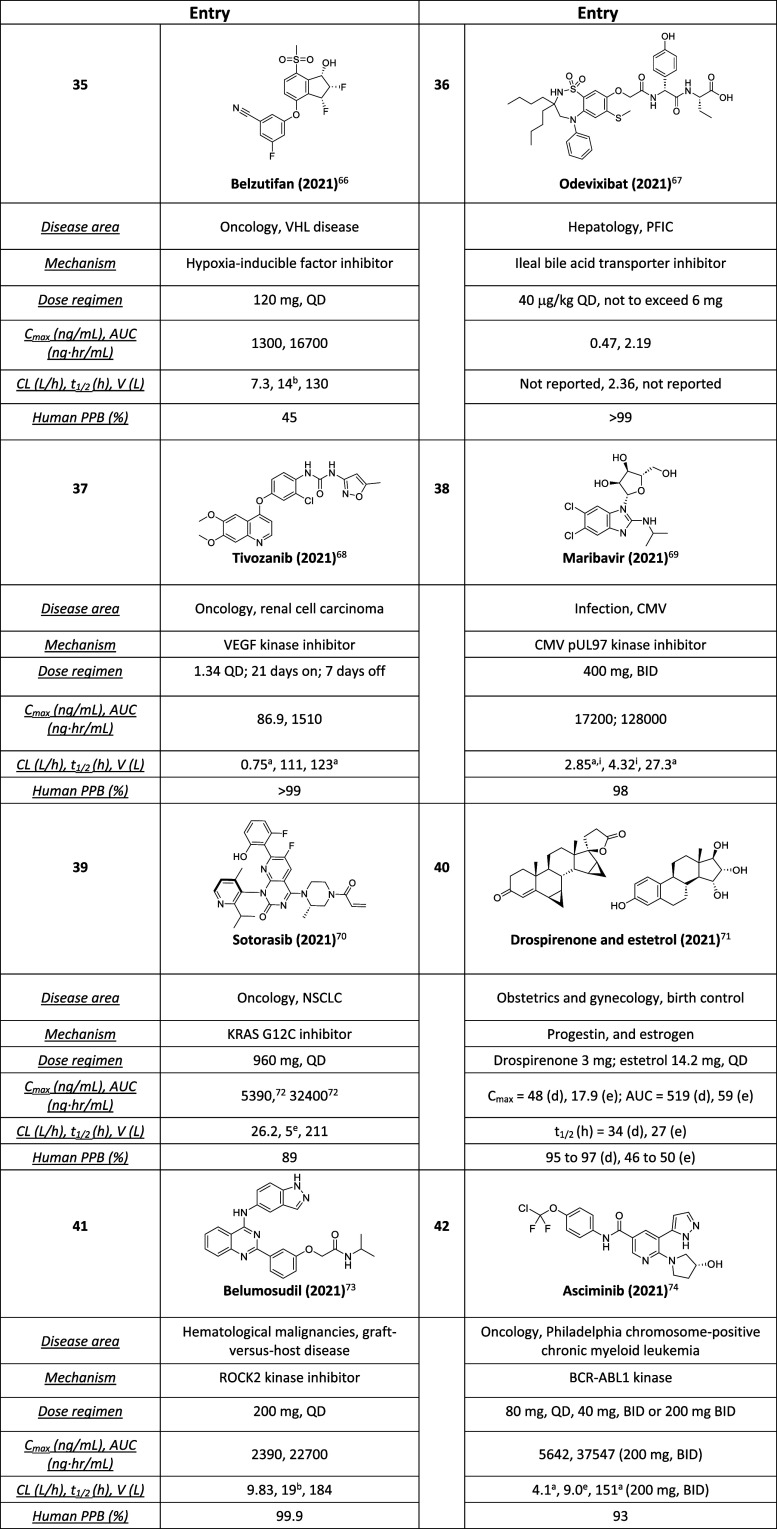

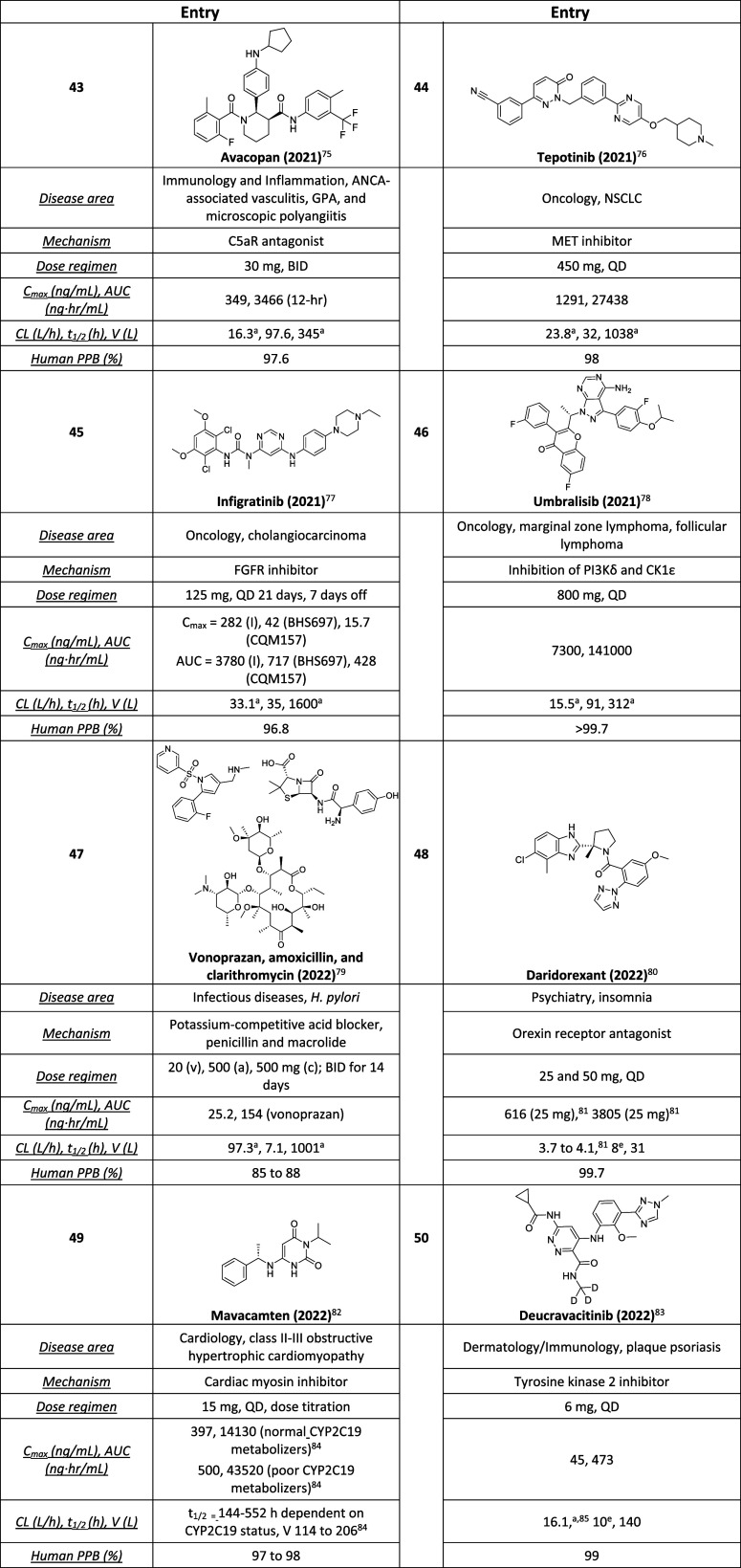

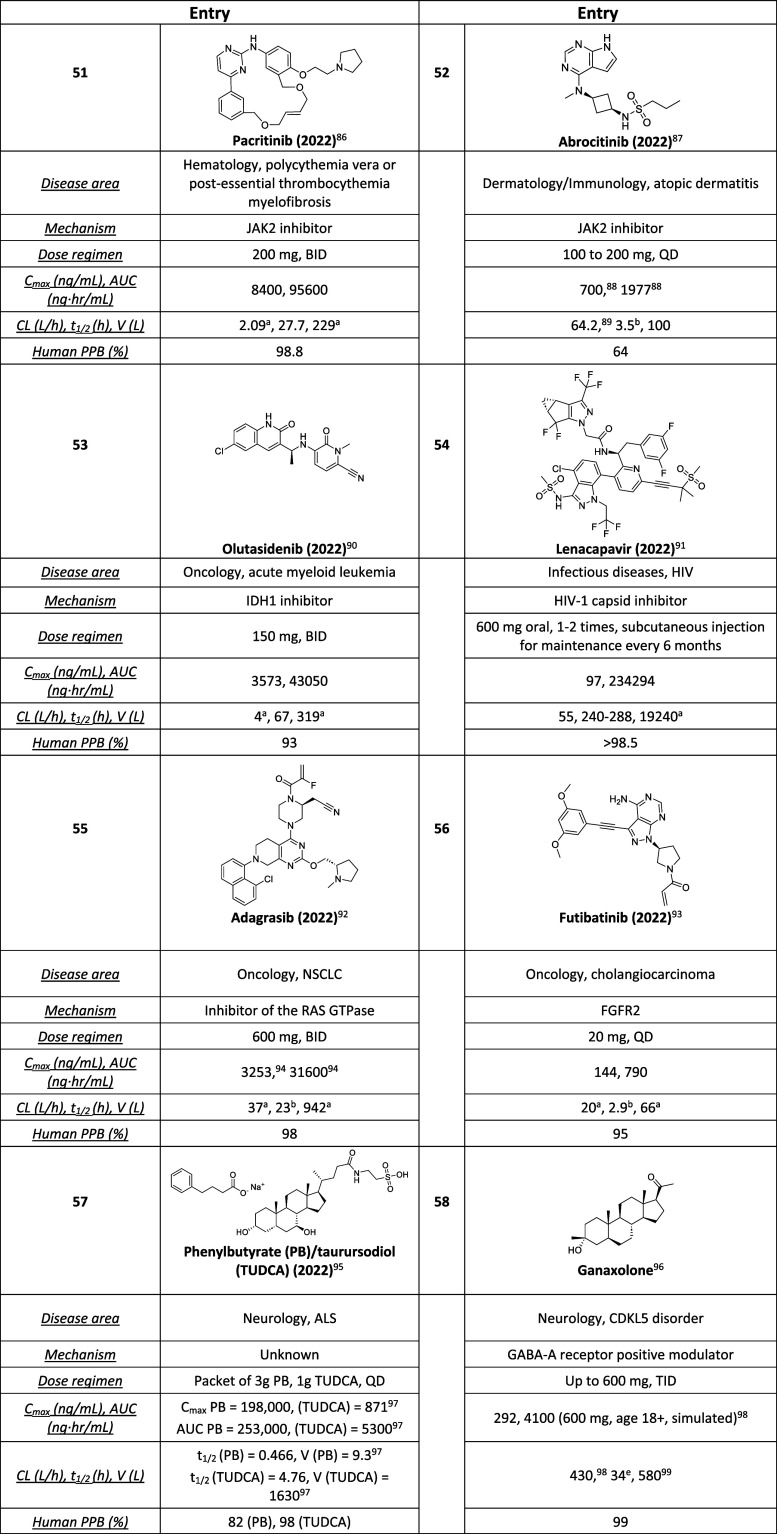

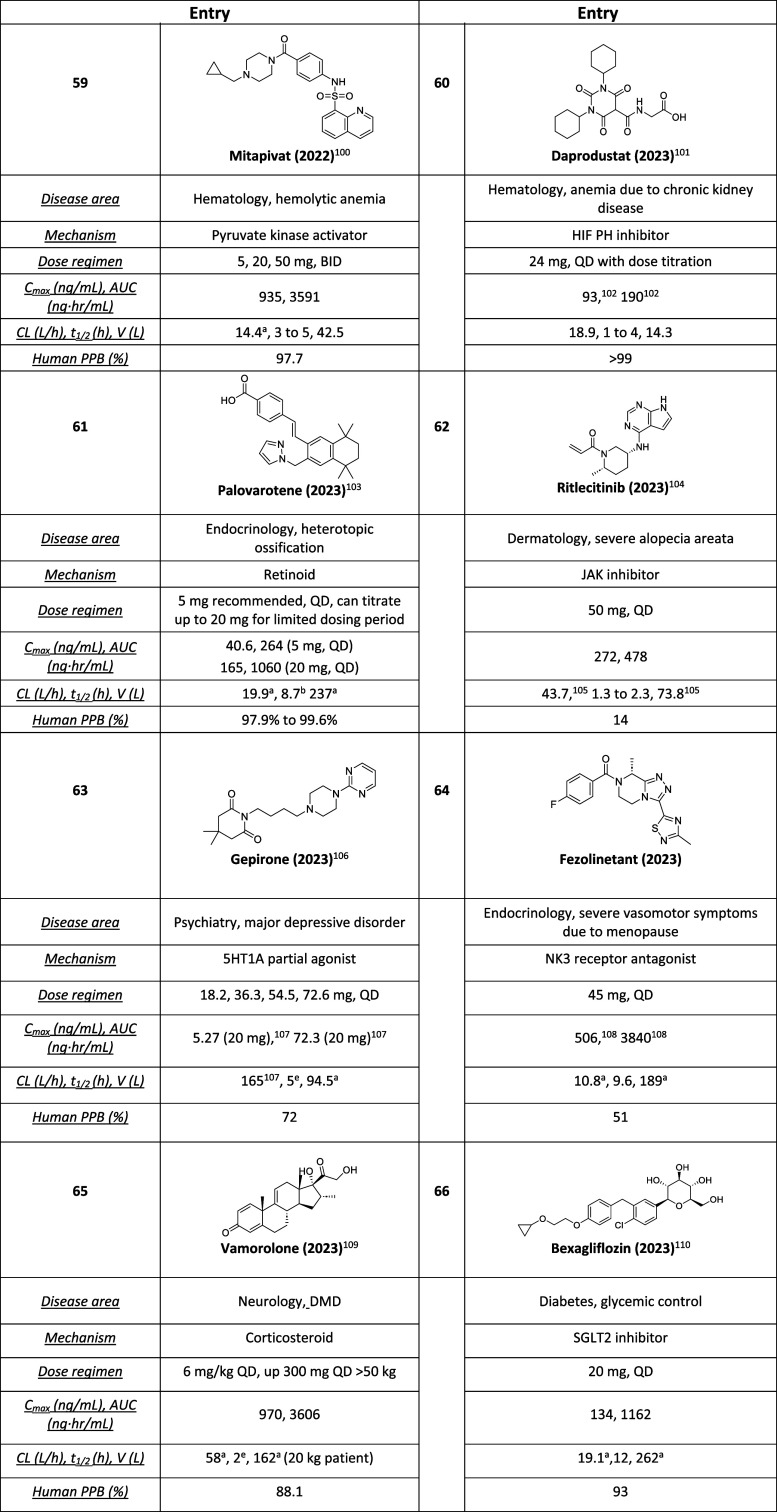

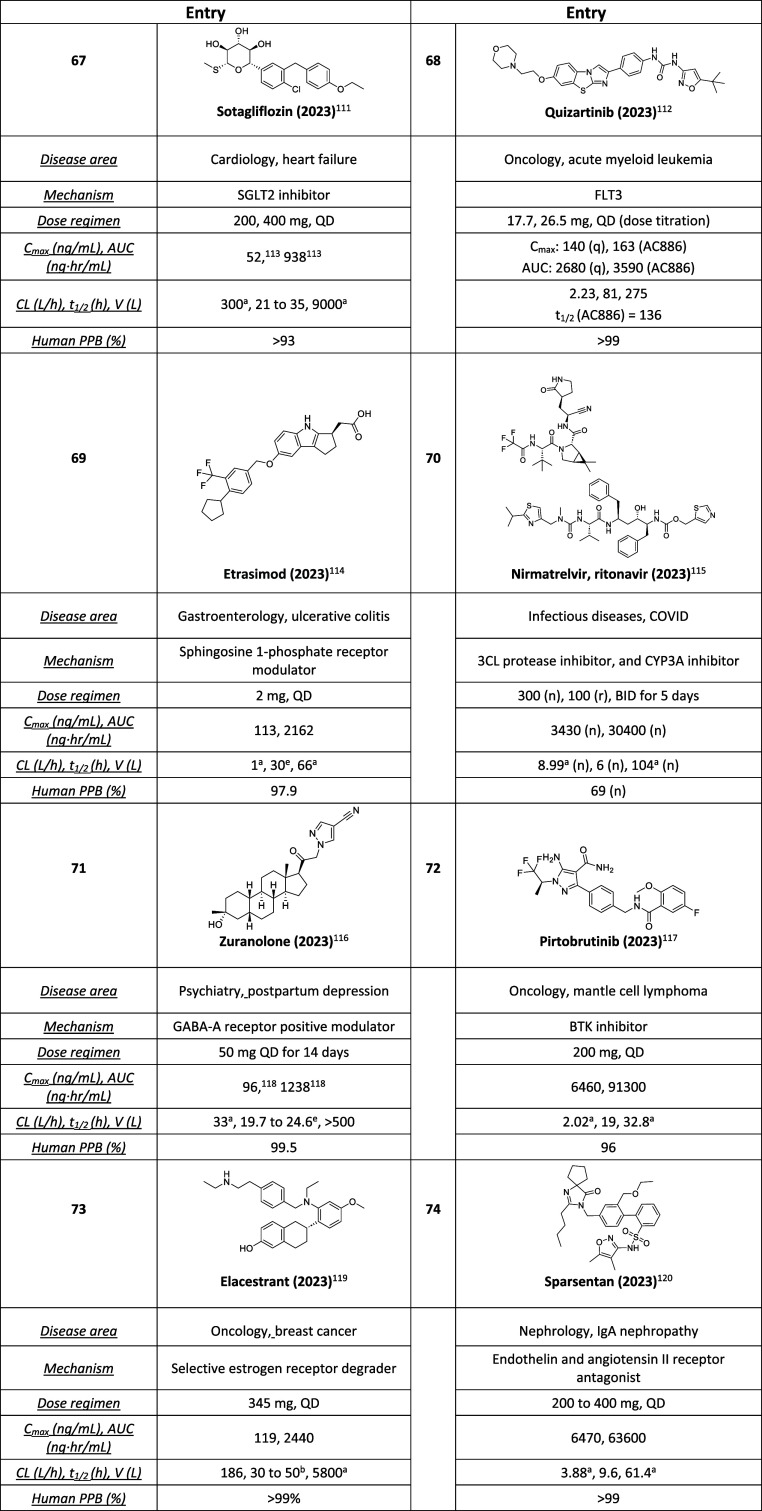

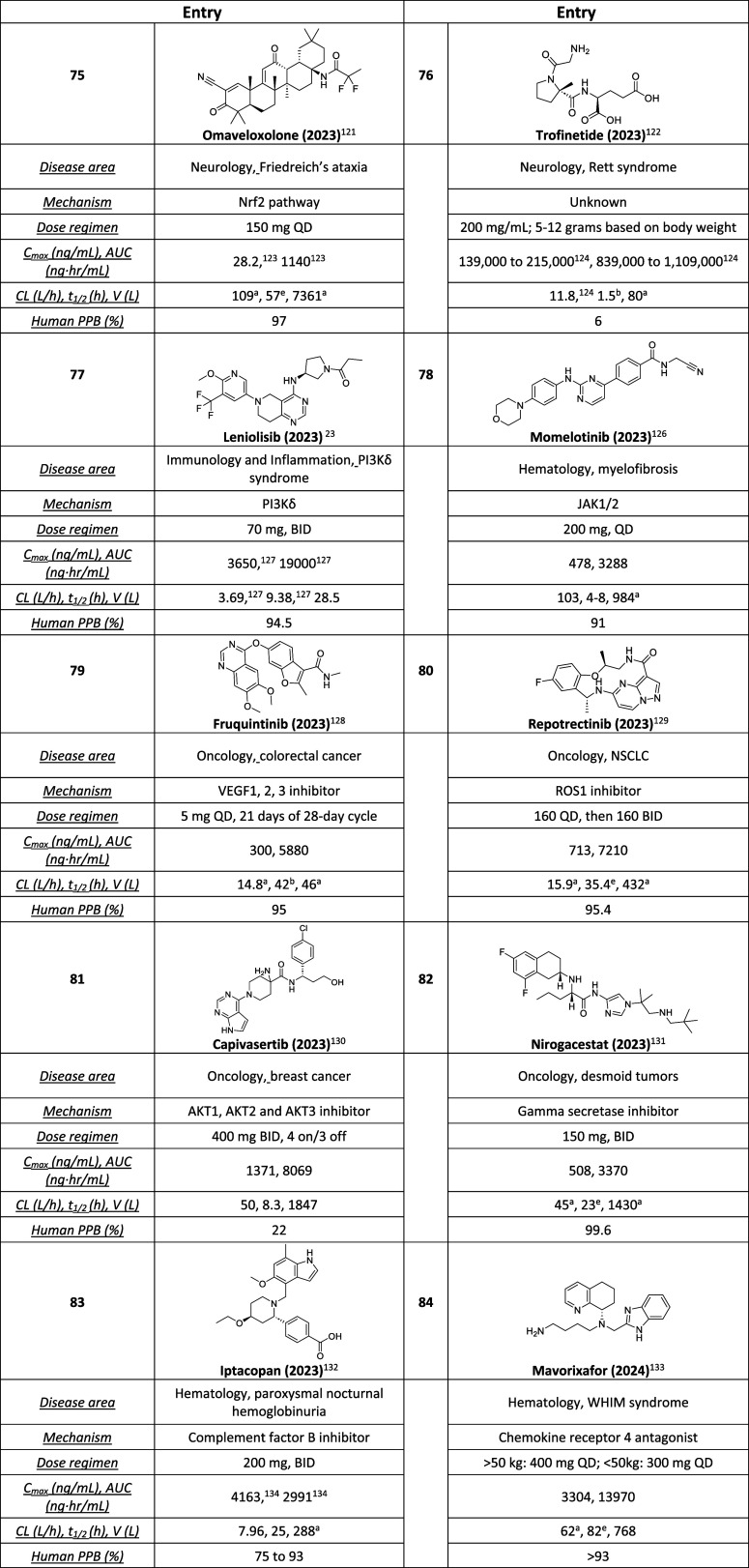

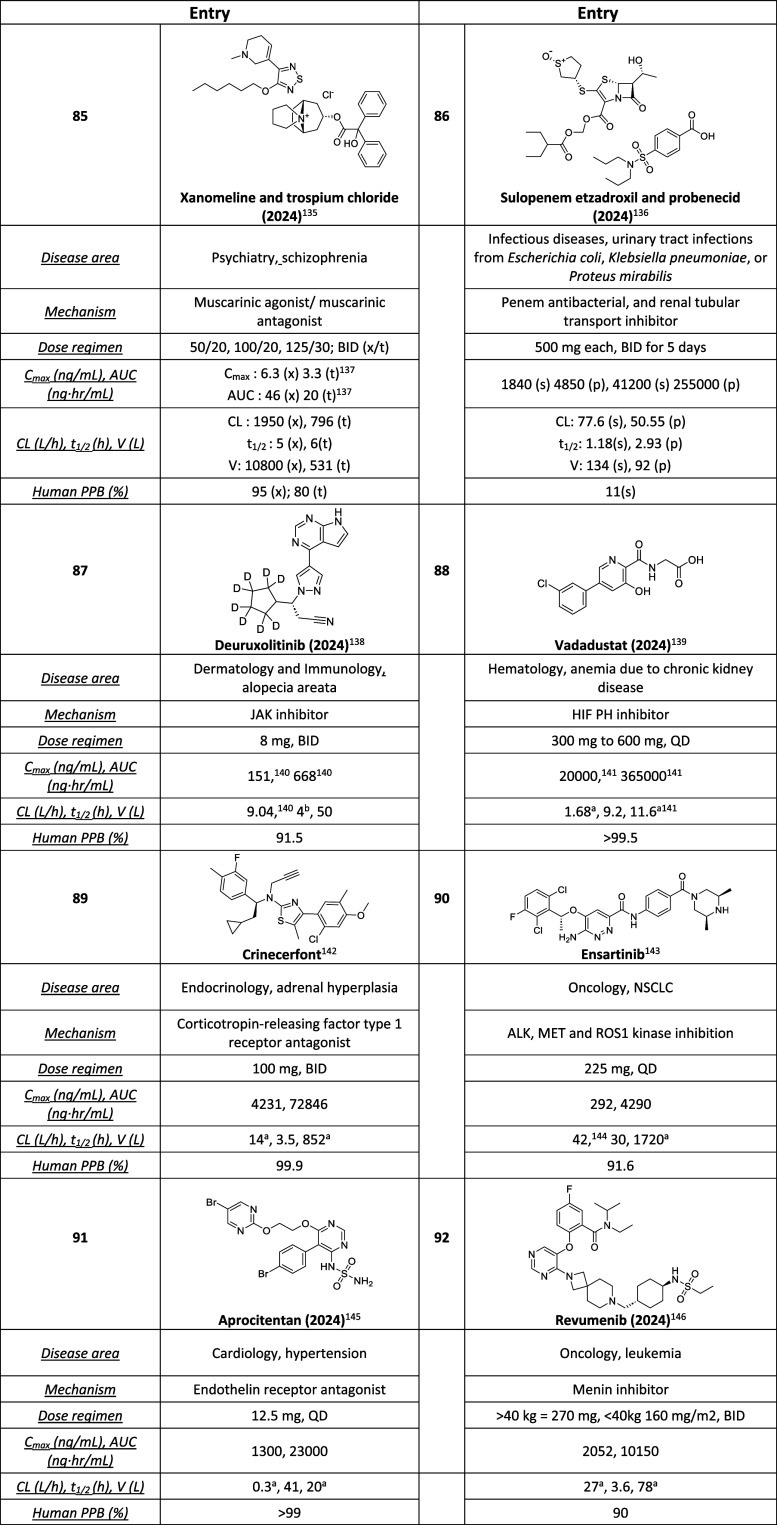

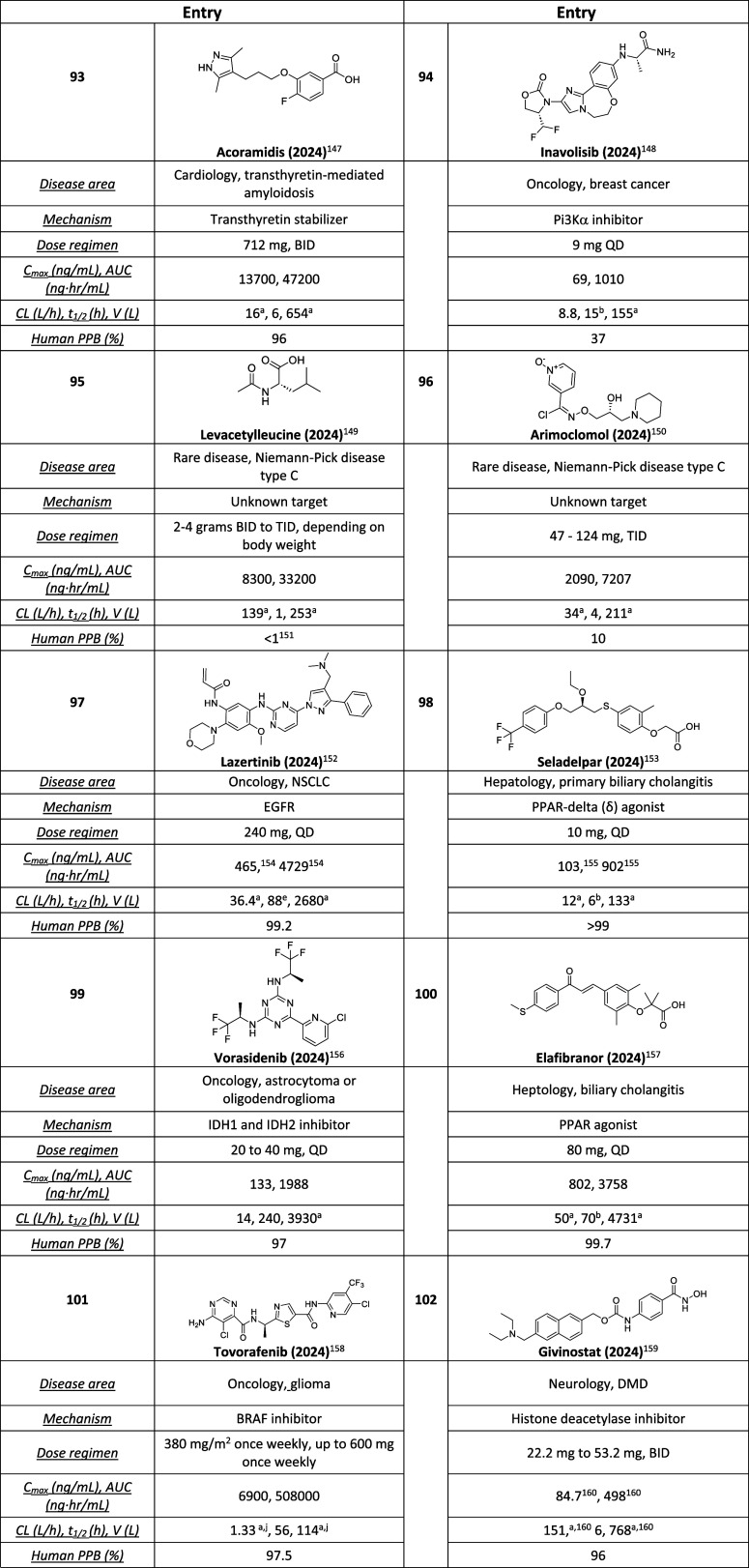

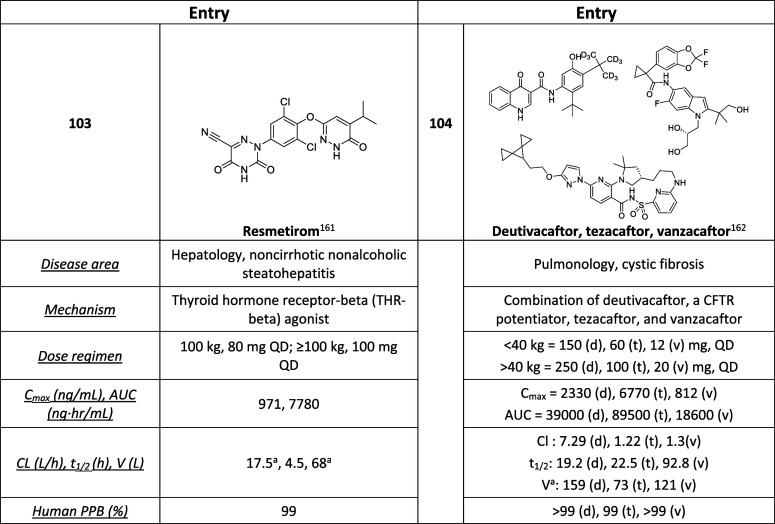
FDA-Approved Small-Molecule Drugs 2020–2024^k^

a Reported as apparent values such as Cl/F or V/F.

b Reported as elimination half-life.

c Reported as mean half-life.

d Reported as effective half-life.

e Reported as estimated mean terminal elimination half-life.

f Half-life based on population pharmacokinetic parameter estimates.

g Reported as mg/kg and converted to 70 kg person in this analysis.

h Because of the low systemic absorption, pharmacokinetic parameters cannot be reliably calculated at the recommended doses.

i Measured in transplant patients.

j Extrapolated to 1.9 m^2^ average male body surface. The data is also provided in the Supporting Information (Data_files).

k For any body weight-driven parameters, assume average adult male: 1.9 m^2^ and average adult female: 1.6 m^2^. PPB = plasma protein binding. Other abbreviations can be found in the section at the end of the manuscript.

In the first part of the analysis, the dosing frequency of each drug was analyzed by approved dosing frequency (TID, BID, QD, and < QD). Figure 2 illustrates that most drugs in this analysis are administered once daily (QD = 67%). The second most common dose frequency is BID (twice daily, 28%) with a few examples of TID (three times daily, 4%) and one example (<1%) of less than QD frequency (Tovorafenib is dosed once weekly, Table 1, Entry 101). In the next part of the analysis, the distribution of dosing frequency according to whether a compound was a first-in-class drug (FIC) or had an Orphan Drug Designation (ODD) was performed. FIC drugs are drugs that produce a novel pharmacologic effect compared to currently approved drugs. Each year, the FDA reports on new drug therapy approvals and highlights those drugs which are considered FIC.^162–166^ The analysis demonstrated that more FIC drugs are approved with either BID or TID dose regimens (50%), as compared with non-FIC drugs (19%) (Figure 3a). This observation may potentially be due to the urgency that sponsors have to be first on the market for a new mechanism. It may be more important to get these novel medicines into the clinic and understand the efficacy in patients, rather than spend a significant amount of time identifying an optimized molecule for a low-frequency regimen. In this regard, a recent report indicated that FIC drugs capture ∼60% market share on average.^167^ Drugs that enter later on the market in the same class will need to differentiate, which can be efficacy, safety, route of administration, or dose frequency.

**Figure 2:**
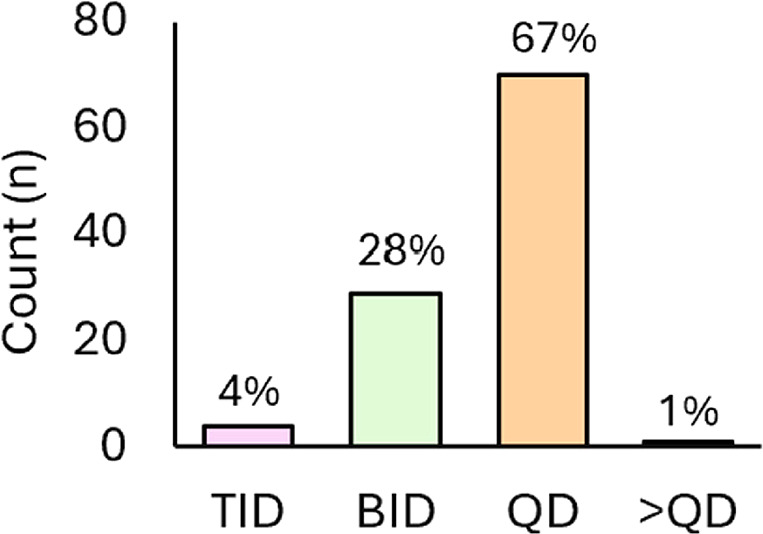
Small-molecule FDA-approved drugs (2020–2024) distributed by approved dose regimen.

**Figure 3:**
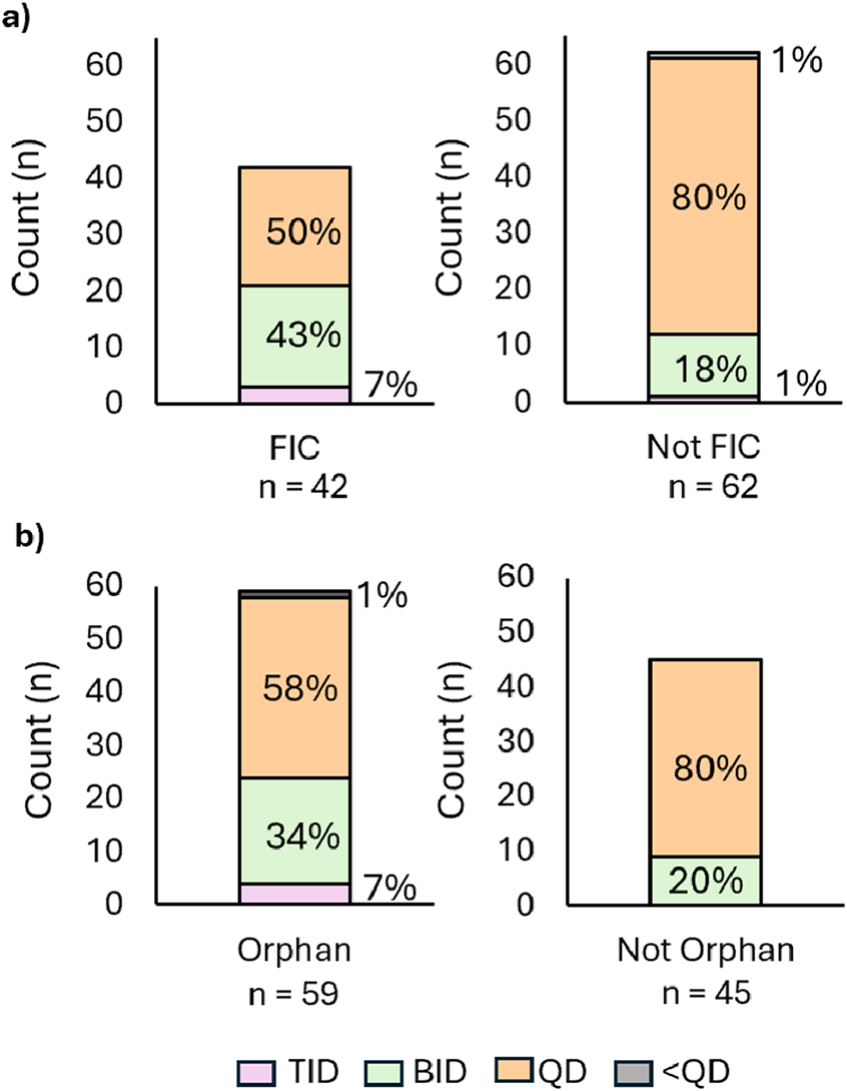
(a) FDA-approved drugs (2020–2024) distributed by dose regimen and FIC designation. (b) FDA-approved drugs (2020–2024) distributed by dose regimen and Orphan Drug Designation.

Another question that was addressed was whether compounds that were developed for rare diseases, such as ODD, were more likely to have BID or TID dosing regimens compared to more prevalent diseases. ODD may be given to potential drugs that meet certain criteria including having less than a 200,000 patient population.^168^ This designation provides certain incentives to sponsors, such as tax breaks, potential additional market exclusivity, and user fee exemptions. The distribution of dosing frequency based on ODD is illustrated in Figure 3b. Based on the analysis, drugs that have ODD are more frequently BID or TID drugs compared to nonorphan drugs (41% vs 20%). An explanation for this observation may be that for rare diseases, patient need and lack of other viable treatment options may support BID or TID dosing. Bringing a safe compound quickly to the market may be more important to reach these patient’s urgent clinical needs, especially if the path to discovering and developing a QD drug is viewed as longer and riskier to optimize. For large, prevalent diseases, QD dosing may be preferable to remain competitive, where other options and existing standard of care may already exist.

The next analysis that was performed was an examination of the maximal daily dose (or the recommended daily dose). Two different analyses were performed on these data. In the first analysis, the total recommended or maximal daily dose was analyzed independent of the dosing frequency or duration. This analysis is illustrated in Figure 4a. Of the 116 drugs (including combination partners), the median recommended or maximal daily dose was 137 mg, with an average dose of 450 mg. For the second analysis, those drugs which were taken less than 14 days in total, had weekly dosing regimens, or had dosing holidays were excluded (*n* = 99 drugs and combination partners, Figure 4b). In this analysis, the median recommended or maximal daily dose was 106 mg, with an average dose of 441 mg.

**Figure 4:**
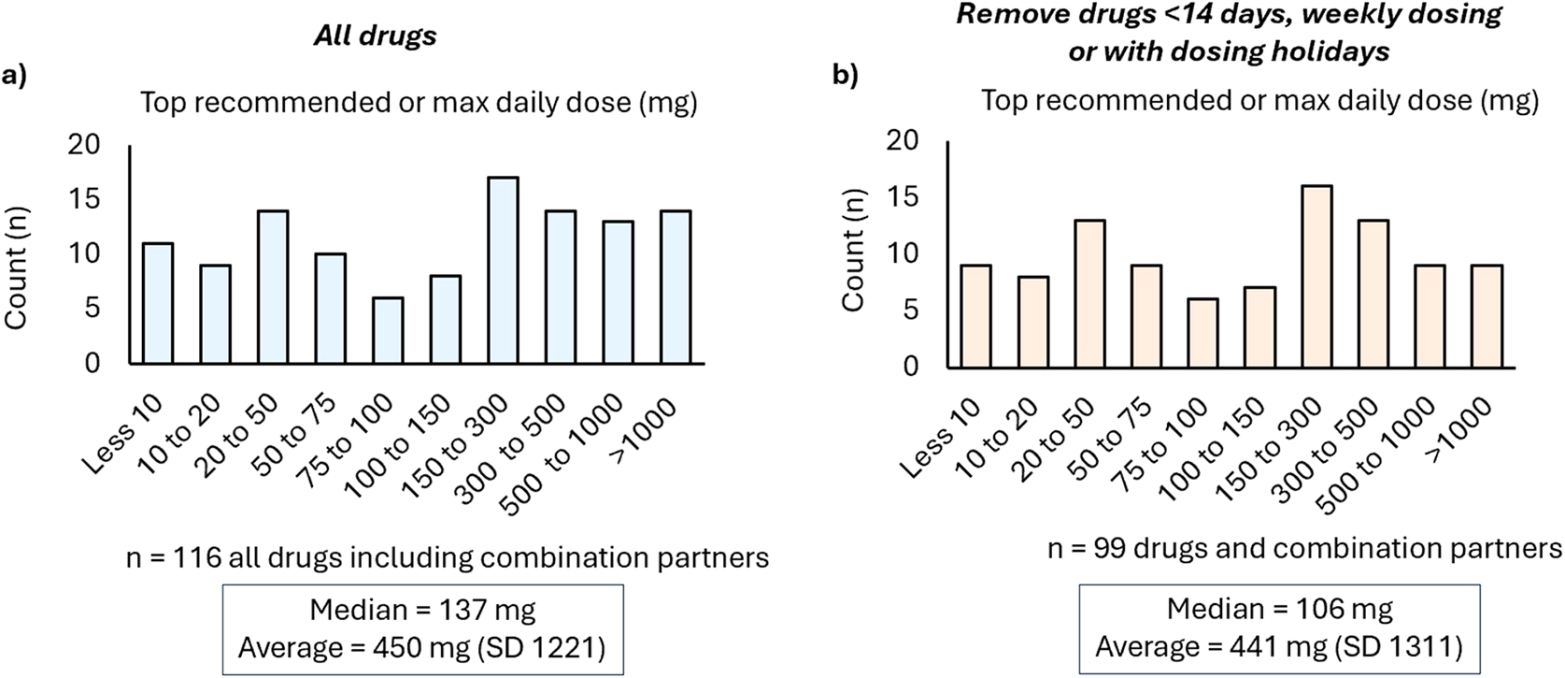
(a) Small-molecule FDA-approved drugs (2020–2024) distributed by maximum daily dose. (b) Small-molecule FDA-approved drugs (2020–2024) distributed by maximum daily dose, excluding drugs dosed less than 14 days in duration or given with dosing holidays.

Parsing these data even further, the daily doses for drugs that were FIC or ODD were examined and compared to those that were not. Table 2 illustrates that FIC drugs (*n* = 42) have a higher median daily dose (200 mg) compared to non-FIC drugs (*n* = 62, median of 122 mg). For ODD, the differences were even more distinct, with a median daily dose of 200 mg for drugs with the ODD (*n* = 59) vs 50 mg for nonorphan (*n* = 45).

**Table 2:** Recommended Daily or Maximal Dose for Oral Drugs (2020–2024) Based on FIC or Orphan Drug Designation^a^

Property	FIC	Non-FIC	Orphan	Non-Orphan
Median daily dose (mg)	200	122	200	50
Mean daily dose (mg), (SD)	848 (1978)	268 (383)	620 (1631)	347 (725)
Count (n)	42	62	59	45

a The total dose included those drugs with combination partners. SD = standard deviation.

Where available in the label or other regulatory documents, selected human pharmacokinetics were collected and tabulated at the recommended daily or maximal approved dose (Table 3). Table 3 summarizes the 25% quartile, median, and 75% quartile for *C*_max_, AUC, V_D_, CL, and *t*_1/2_ for the recommended daily or maximal approved dose where available. If the recommended daily dose pharmacokinetics were not available, the nearest dose from a healthy volunteer study was chosen instead. Based on this data, the median *C*_max_ was 500 ng/mL, and AUC was 3769 ng·h/mL. The median V_D_ was 235 L, with a median CL of 19 L/h. As expected, since most drugs are QD administration (67%, Figure 2), the median half-life (*t*_1/2_) was long at 14.8 h. These parameters are useful to help gauge compounds in early discovery against human dose predictions, and some methods to do this will be discussed later in the manuscript. As can be seen in Table 1, there are approved drugs in this set with very high exposures, high clearances, and short half-lives. A few examples of these will be discussed later in the manuscript.

**Table 3:** Human Pharmacokinetics of Small-Molecule FDA-Approved Drugs (2020–2024)

Range	*C*_max_ (ng/mL)	AUC (ng·h/mL)	*V*_D_ (L)^a^	CL (L/h)^a^	*t*_1/2_ (h)
25% quartile	96	911	94.5	8.8	6
Median	500	3769	235	19	14.8
75% quartile	2300	23,000	984	53.5	30.8
Mean (SD)	7731 (43,458)	53,795 (192,238)	1296 (2757)	74 (212)	30.5 (48.17)
Count	117	114	97	113	104

a Reported as apparent values such as Cl/F or V/F, SD = standard deviation.

Several recent articles have been written about the role of PPB and how chemists should apply and use this information. For example, a recent article by Webborn et al. suggested a number of misconceptions of PPB and offered insight into the correct use of total and free drug, such as estimating concentrations for efficacy and toxicity studies, and linking to a pharmacological effect.^169^ In this regard, it was of interest to understand the range of PPB across this set of drugs. In most cases, the human PPB was reported in the label or in other regulatory documents. In addition to the parent drugs, combination partners and active metabolites (where reported) were also included (*n* = 115). The analysis demonstrated that the majority of drugs have a reported PPB ≥95% (59% of all drugs), with 29% having reported values of >99% binding to human plasma protein (Figure 5). In contrast, only a small proportion (<18%) is below 70%.

**Figure 5:**
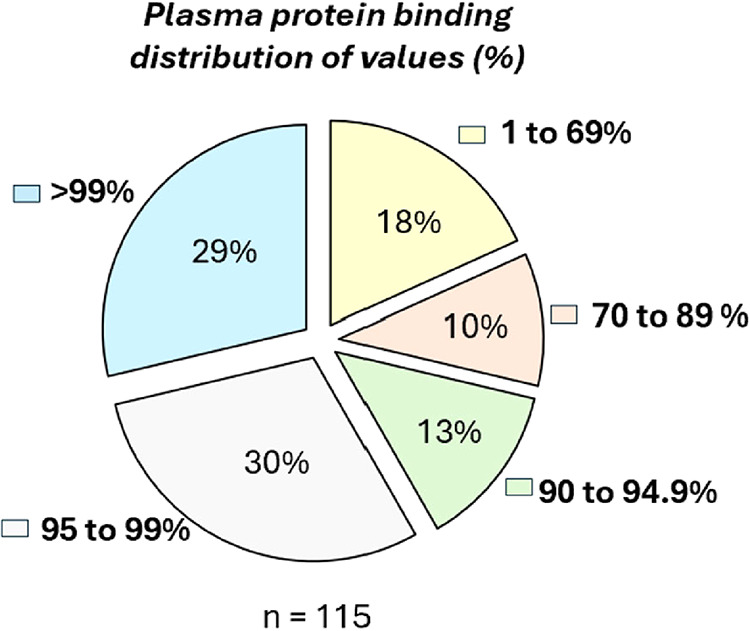
Small-molecule FDA-approved drugs, combination partners, and active metabolites (where reported) (2020–2024) distributed by % human PPB.

Oral bioavailability is also a key pharmacokinetic parameter that drives the human dose and exposure. This parameter is not as widely reported on labels or in other readily accessible regulatory documents and hence could be easily obtained for only 32 reported examples. From this set of 32 examples, there appeared to be a wide distribution of oral bioavailabilities (Figure 6). In one extreme example, xanomeline (Table 1, Entry 85) (which is part of a combination partner with trospium) is reported to have ∼1% oral bioavailability due to extensive first pass metabolism.^170^ Other examples of relatively low bioavailability include relugolix (12%, Table 1, Entry 22),^42^ nirogacestat (19%, Table 1, Entry 82),^130^ and sotagliflozin (25%, Table 1, Entry 67).^111^

**Figure 6:**
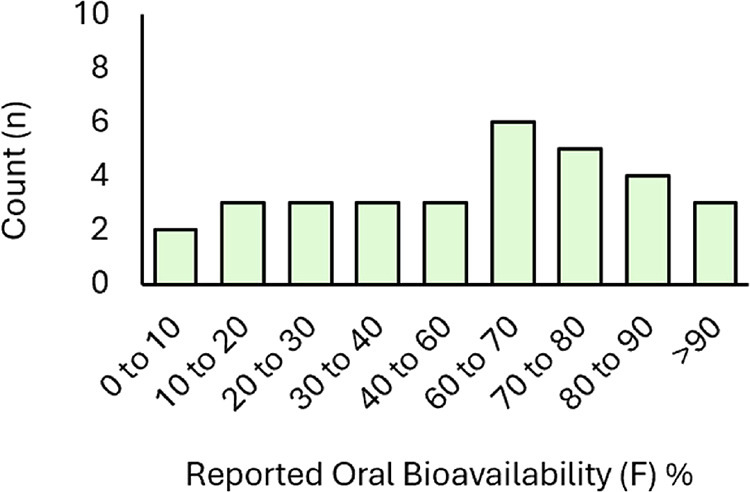
Small-molecule FDA-approved drugs and combination partners (2020–2024) distributed by reported bioavailability for *n* = 32 reported examples.

In addition to pharmacokinetic properties, selected safety and warning data that was included on the labels were also analyzed for these recently approved drugs. Black box warnings are added to the label as the most serious level of warning provided by the FDA.^171^ Of the approved drugs in this analysis, 22% of these drugs had black box warnings (Figure 7a). Another level of warning included on the label is contraindications. Figure 7b illustrates that 42% of these drugs have a contraindication on the label. No attempt was made to categorize and quantify the contraindications; however, it appears that a common contraindication across many of these drugs is hypersensitivity to the drug itself. Finally, Figure 7c illustrates the percentages of compounds that have a warning based on QT prolongation (16%). When these approved drugs are categorized by disease indication (Figure 8), oncology has ∼14% black box warnings for all oncology approvals compared to 28% for neuroscience compounds. Proportionally, cardiovascular drugs, autoimmune drugs, and renal drugs have more black box warnings; however, the overall number of approved drugs in each of these categories is relatively small. Thus, it is not possible to draw any conclusions that those specific disease areas are more likely for a drug to have a black box warning. Of the black box warnings, many contain cardiovascular warnings, including 2 with QT prolongation and Torsades de pointes, which are also included in Figure 7c. Four of the drugs contain warnings about embryo-fetal toxicity, and two contain warnings about suicidal thoughts. In addition, another common warning was for serious infections, which is indicated for four of the drugs. Several drugs have multiple warnings, such as serious infections and cardiovascular events.

**Figure 7:**
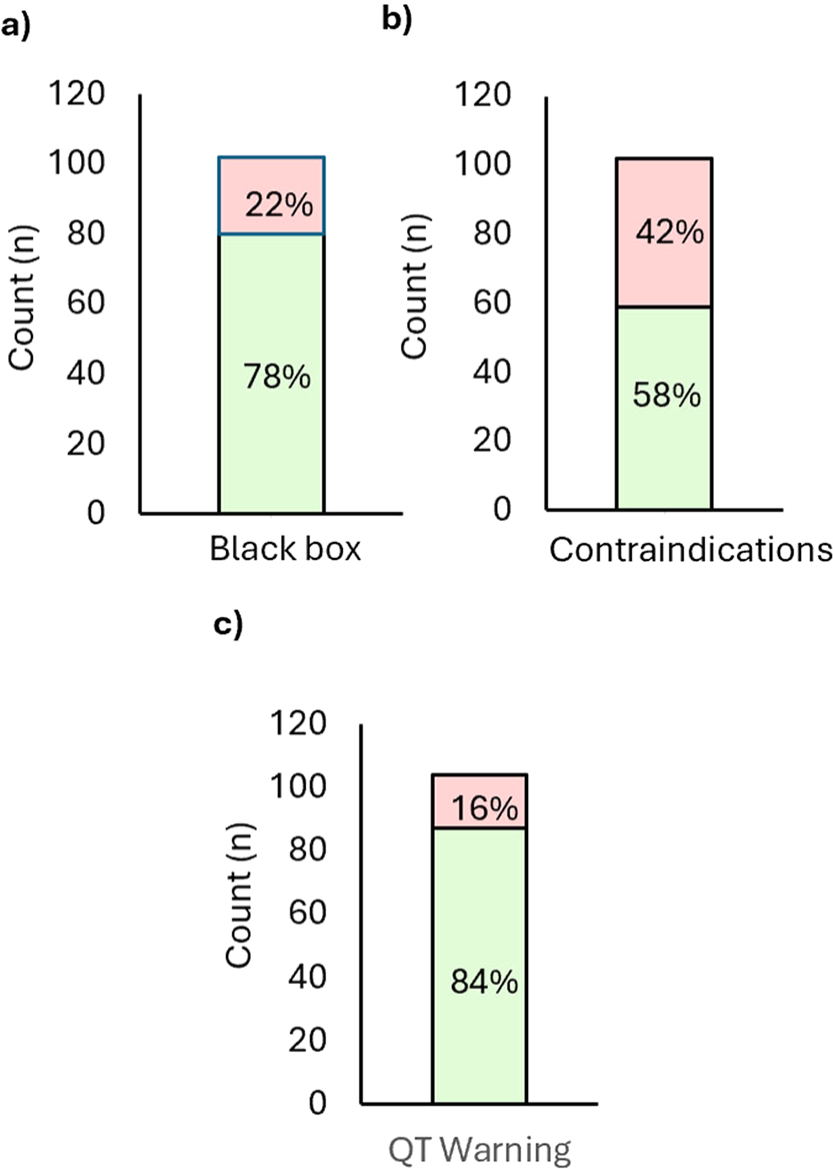
(a) Small-molecule FDA-approved drugs (2020–2024) by black box warnings. (b) Small-molecule FDA-approved drugs (2020–2024) by contraindication. (c) Small-molecule FDA-approved drugs (2020–2024) by QT warnings.

**Figure 8:**
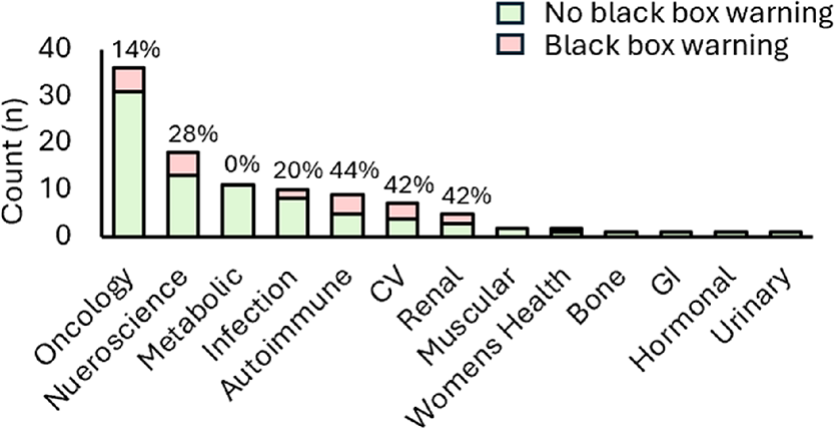
Small-molecule FDA-approved drugs (2020–2024) by black box warnings according to therapy area.

In addition to safety data, the primary route of metabolism, DDI, and reported active metabolites were extracted from the labels or other readily accessible regulatory documents. For the primary route of metabolism, it should be noted that in some cases, more than one primary source of metabolism was listed on the label. In those cases, all primary sources were counted for those compounds. This analysis is presented in Figure 9. Based on the analysis, 46% of these compounds are primarily metabolized by CYP3A4, followed by CYP2C8 (8%), CYP2C9 (6%), and CYP1A2 (6%). Among the glucuronosyltransferase enzymes, UGT2B7 (6%), UGT1A9 (5.2%), UGT 1A3 (2.2%), and UGT2B17 (2.2%) were the most widely noted in the approved label.

**Figure 9:**
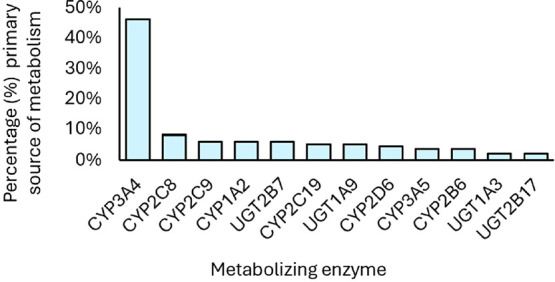
Small-molecule FDA-approved drugs (2020–2024) by the major metabolizing enzyme. It should be noted that in some case, more than one primary source of metabolism was listed on the label. In those cases, all primary sources were counted for those compounds. *n* = 134 drugs, combination partners and active metabolites.

The next analysis that was conducted was to understand the distribution of DDIs reported on the label. For each label, there may be various levels of DDIs, such as contraindicated (the most serious), avoid, adjust dose, and monitor as examples of the warning provided. This may be even further stratified according to the strength of the potential other drug as the perpetrator or victim (e.g., strong or moderate). For simplicity, this analysis did not stratify all of the possible scenarios. The DDI analysis is shown in Table 4. Based on the analysis, the largest category of DDIs is CYP3A4 inducers (62/104), followed by CYP3A4 inhibitors (48/104) and then CYP3A4 substrates (30/104). Outside of CYP enzymes, P-gp substrates (22/104) and BCRP substrates (12/104) were among the most common.

**Table 4:**
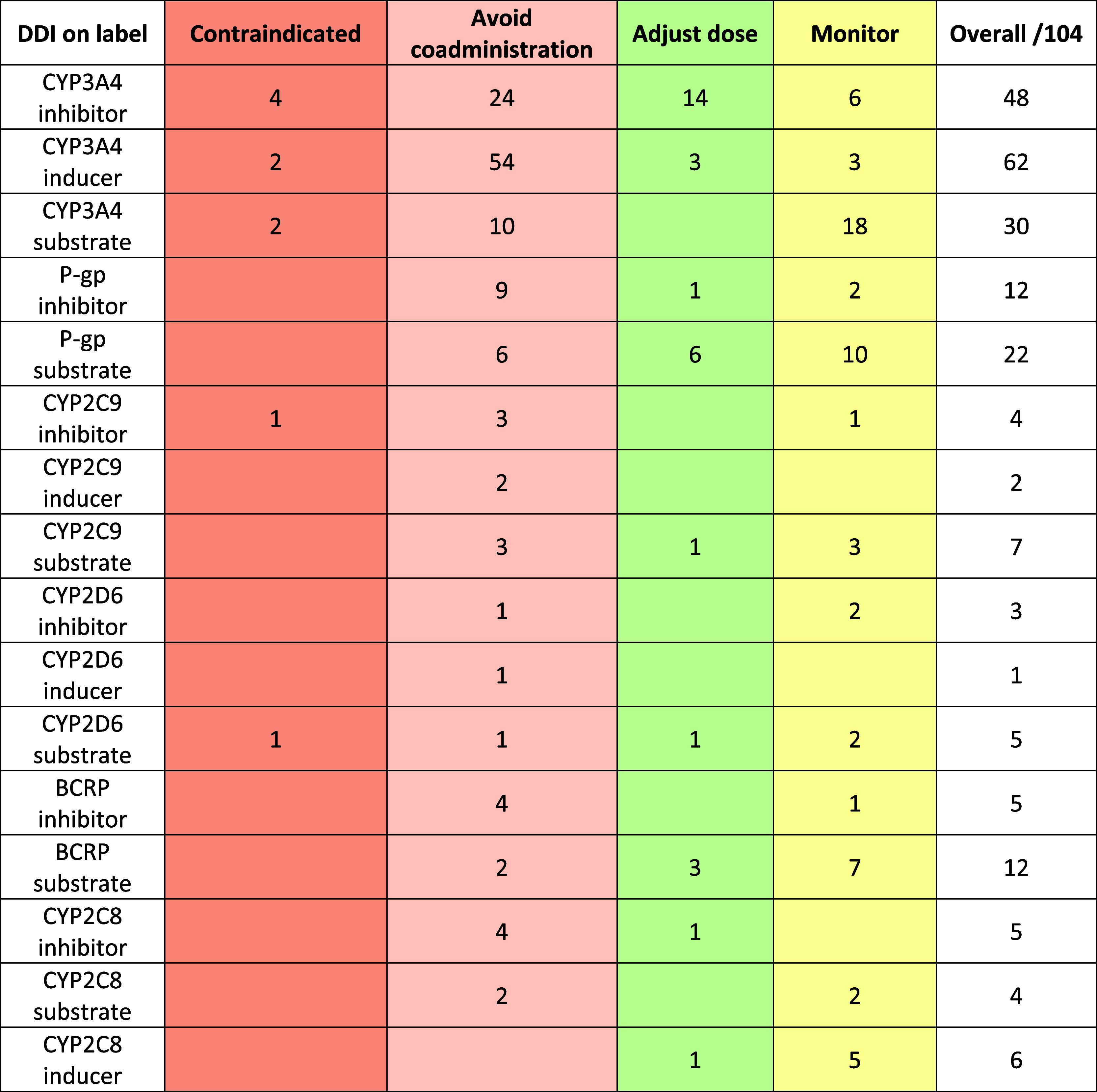
Small-Molecule FDA-Approved Drugs (2020–2024) Distributed by Number of DDI Label Warnings for Any Cases (Strong, Moderate, or Weak)

### Active Metabolites

Of the 104 drugs analyzed, at least 14 were reported to have active metabolites. An active metabolite in a drug is defined as a metabolite which may bind to the therapeutic target of interest or interact with other receptors to have potential side effects.^172^ Ideally, such metabolites can be characterized and qualified in nonclinical toxicity studies if they are formed in those species. In cases where they are not (e.g., disproportionate metabolites), these metabolites may need to be individually tested in chronic toxicology studies (whether they are pharmacologically active or not).

*N*-dealkylation is a common pathway of metabolism through CYP-mediated oxidation and, in some cases, can lead to active metabolites. Figure 10 highlights several of the examples from Table 1 that reported an active metabolite due to *N*-dealkylation. In Figure 10, selumetinib is illustrated, which is primarily metabolized by CYP3A4, leading to *N*-desmethyl selumetinib. Although *N*-desmethyl selumetinib is <10% of selumetinib levels in human plasma, it is more potent than the parent compound and ultimately contributes between 21% and 35% of the combined pharmacologic activity of a selumetinib dose.^22^ A further example is ripretinib that loses the *N*-methyl group to give DP-5439, which has approximately similar potency to ripretinib.^173^ Ripretinib is metabolized predominately by CYP3A4, but it also has contributions from CYP2C8 and CYP2D6. Mobocertinib has two active metabolites, both of which are *N*-dealkylation products but are in two different areas of the parent compound.^174^ These metabolites are reported to have similar in vitro activity to mobocertinib.^174^ Mobocertinib is primarily metabolized by CYP3A4.^63^ Infigratinib is a similar example which is primarily metabolized by CYP3A4,^77^ giving rise to two active metabolites that are equally potent to the parent.^175^ The final example in Figure 10 illustrates deucravacitinib, which is metabolized by CYPA12 to give BMT-153261.^83,176^ This example is also of note since deuteration of the *N*-alkyl group was intentionally designed to minimize *N*-dealkylation that would result in a potent and nonspecific kinase inhibitor.^177^

**Figure 10:**
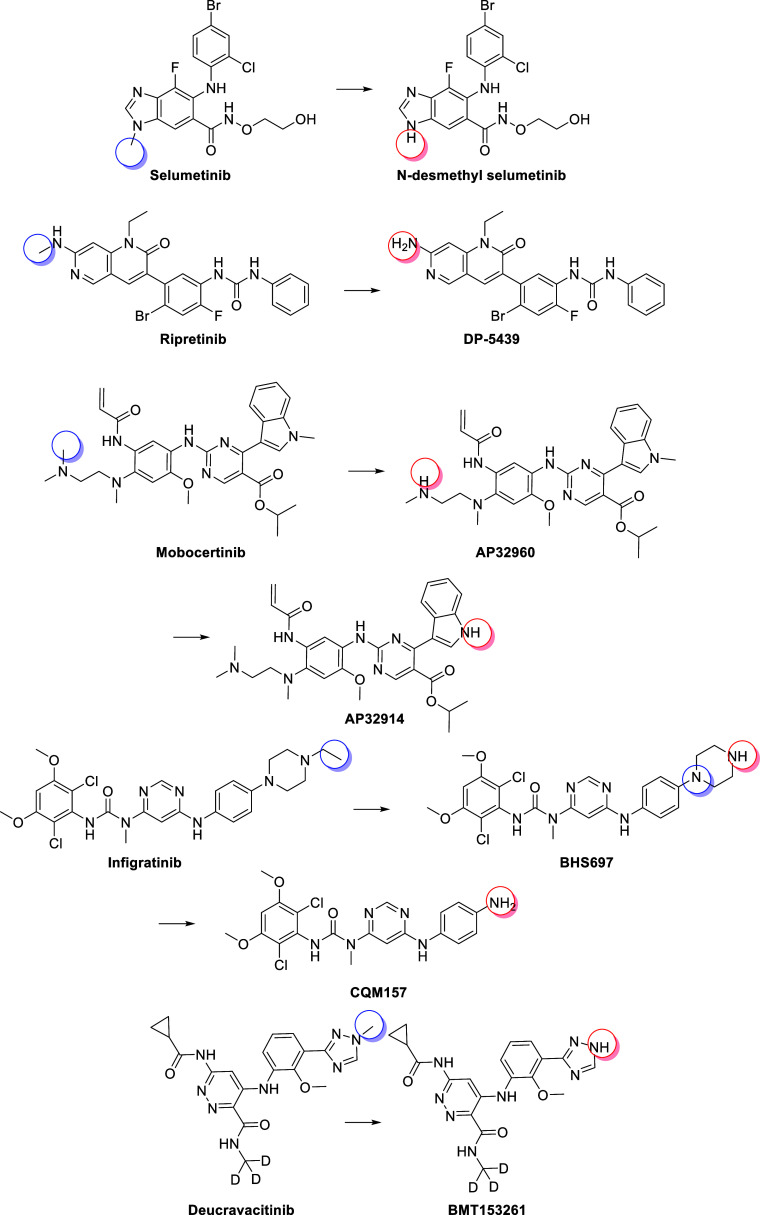
Examples of *N*-dealkylation giving rise to active metabolites.

Additional small-molecule FDA-approved drugs with active metabolites that do not involve *N*-dealkylation are shown in Figure 11. In the first example, fexinidazole is metabolized to two different active metabolites, fexinidazole-M1 and fexinidazole-M2, arising from mono- and dioxidation of the sulfur group.^178^ The AUC levels of M1 and M2 are substantially higher than parent (11- and 34-fold higher, respectively).^57^ The next example in Figure 11 is avacopan, which has an active metabolite formed by CYP-mediated hydroxylation of the para-substituted aromatic methyl group.^179^ Abrocitinib has two oxidative metabolites that are considered active metabolites, both on the propyl side chain.^89^ Gepirone has a more complicated metabolic pathway, which yields two active metabolites, 3′–OH-gepirone and 1-(2-pyrimidinyl)-2-piperazine (1-PP).^180^ Quizartinib also possesses an oxidized active metabolite, in this case oxidation on the *tert*-butyl position resulting in AC886.^181^ Momelotinib forms an active metabolite (M21), which is ∼40% the activity of the parent compound.^182^ This metabolite is formed via aldehyde oxidase, and the AUC ratio is 1.4 to 2.1 compared to the parent.^112^ A final example is elafibranor, which gives rise to a metabolite where the styrene moiety is reduced to give GFT1007.^183^

**Figure 11:**
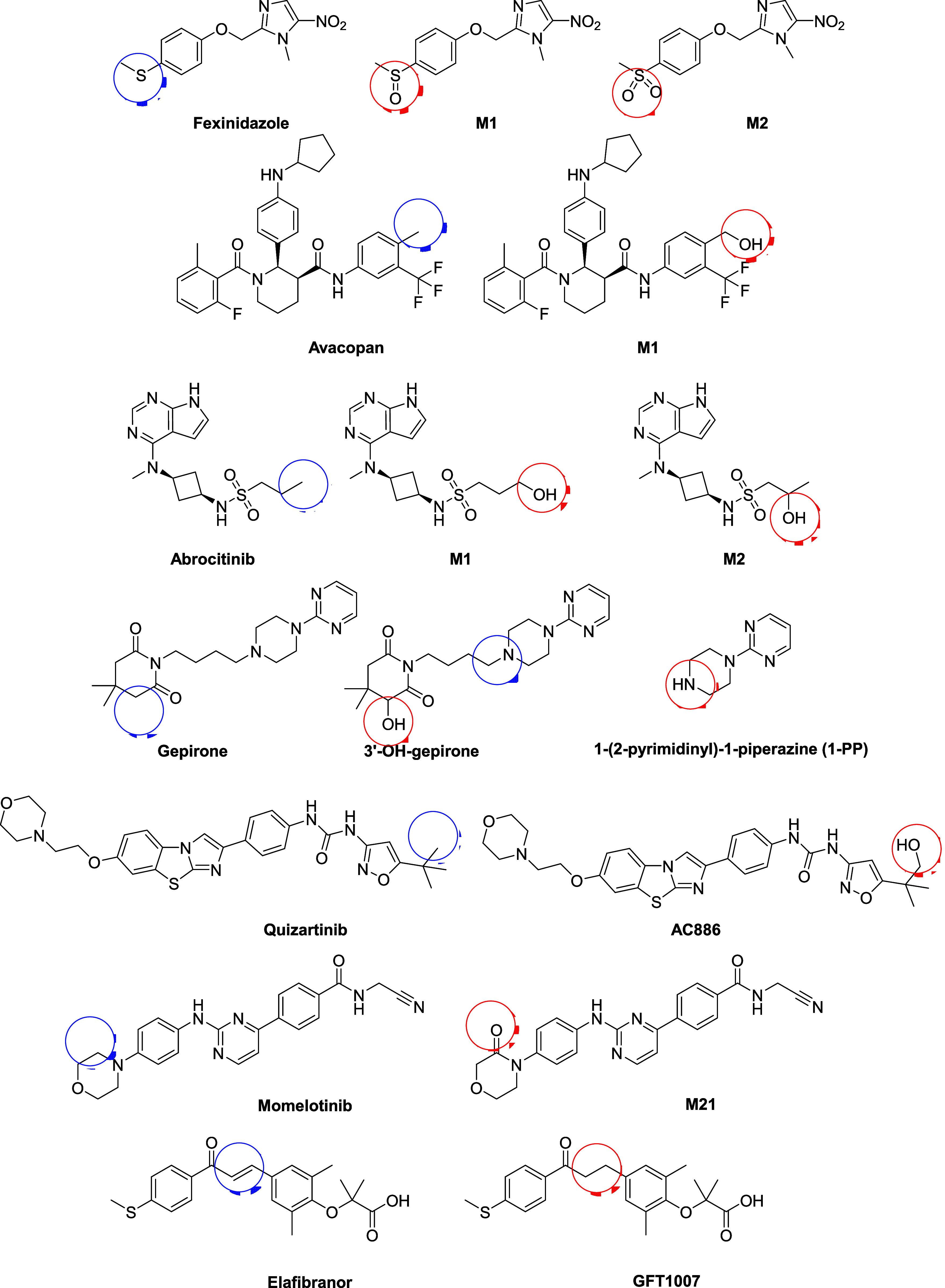
Examples of active metabolites.

A final example is a complex case study whereby ozanimod is metabolized to multiple active metabolites (Figure 12). Ozanimod is a sphingosine-1-receptor modulator approved for multiple sclerosis in 2020. The compound has a complex metabolic profile and generates two active metabolites that contribute to the pharmacology.^184^ Among the metabolites generated, a major circulating active metabolite is CC112273, which is also reversibly converted to CC108097. The metabolism of the parent molecule happens predominately through three distinct pathways, which are the following: (a) aldehyde dehydrogenase and alcohol dehydrogenase, (b) CYP3A4 and 3A1, and (c) reductive metabolism by gut microflora. The formation of CC112273 is done by monoamine oxidase B, providing CC108097, which is then subject to aldo-keto reductase 1C1/1C2 and/or 3b- and 11-β-hydroxysteroid dehydrogenase, to reversibly form CC112273.^184^ Understanding this complex metabolic pathway and disproportionate metabolites, supported by human radiolabeled studies, was essential to obtaining regulatory approval.

**Figure 12:**
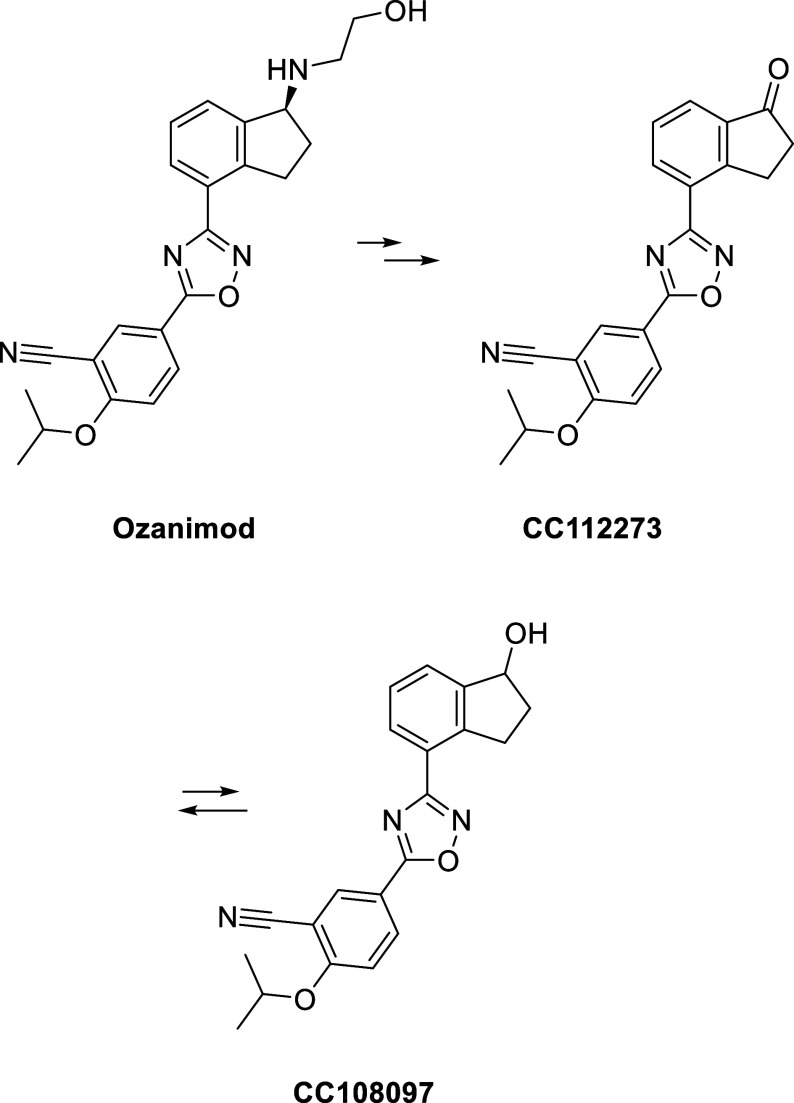
Active metabolites from Ozanimod.

### What Can Chemists Learn from This Analysis?

#### Guiding Principle No. (1) Overly Prescriptive DC Criteria May Inadvertently Stifle the Development of Innovative Drugs That Could Ultimately Serve Patients in Need

As discussed in the introduction, the DC (development candidate) criteria often serve as the North Star of the project team. There can, however, be a tendency to impose strict DC-criteria, which at the surface may make logical sense to reduce risky programs from entering development. That is to say, if the DC criteria are designed in such a way that it increases the probability of clinical success, and conversely decreases the likelihood of expensive late stage failures, those strict criteria may help a sponsor from wasting money on things that are high risk and help them to focus their finite pool of resources to lower risk activities. One example of this may be human daily dose criteria. As mentioned previously, human daily dose has been correlated with DILI. Some early papers in this field of study concluded that daily doses <10 mg are associated with less DILI than those drugs ≥50 mg.^185^ Conceptually, this makes sense as a reduced body burden is likely to reduce potential unwanted toxicities that may be associated with higher exposures of parent drug or metabolites. How often though are approved oral drugs <50 mg daily dose? In this analysis, 34/104 (32%) are at 50 mg or below for their total daily doses. If one excludes the acute drugs (less than 14 days), and the ones which have dosing holidays (presumably due to mechanism-based toxicity), the numbers are similar, with 30/99 (30%). On the other end of the spectrum, 41/104 (39%) have daily doses >300 mg, and when acute and dosing holiday drugs are excluded, it is 31/99 (31%). This is not to say that high dose drugs are preferred or are easy to develop. There are issues that accompany high dose drugs, including manufacturing costs for the increased scale, ability to formulate into a swallowable tablet, and patient tolerability as examples. Nonetheless, there are many examples in this set which will help to provide background and rationale for the type and numbers of drugs that are high­(er) dose, which may have not been developed if a strict view of daily dose requirements was imposed on the team early in the program. More intriguingly, the compounds which are ODD or FIC are often higher daily doses (median = 200 mg) compared to drugs without those classifications (50 mg and 122 mg, respectively). In addition, these categories of drugs are more likely to be BID or TID compared to drugs without FIC or Orphan designation.

Another observation that may fall into the category where overly prescriptive DC criteria may impede innovation is the number of drugs that have relatively high clearance and/or low oral bioavailability. One example is xanomeline (Table 1, Entry 85), which has a reported CL of 1950 L/h (and 1% oral bioavailability as previously reported above). Other intriguing examples include ozanimod (Table 1, Entry 4), which has CL = 192 L/h (and also has a very complex interplay of active metabolites), ganaxolone (Table 1, Entry 58) with a CL = 430 L/h, and sotagliflozin (Table 1, Entry 67) with a CL of 300 L/h.

In addition to these two categories, there were other areas where dogma or a strict view of what a drug would look might have prevented development. Examples include safety warnings, such as QT prolongation, contraindications, and DDIs that were illustrated above. Many approved drugs in this analysis have a warning for DDIs, in particular, CYP3A4 induction (62%) and CYP3A4 inhibition (48%). Profiles such as these may not be commercially viable for a drug that is a late market entry (e.g., a “me-too” drug) or used for a large prevalent disease with frequent comorbidities and comedications but may be acceptable for diseases where patients have no other options and the safety risks can be adequately monitored and justified.

#### Guiding Principle No. (2) Chemists Should Educate Themselves with the Ability to Calculate Early Human PK Properties and Dose Predictions

In the career of most medicinal chemists, they will likely run into the phrase, “That will never be a drug!”. This is an easy thing to speculate on of course because it is almost always a true statement! Throughout the career of a medicinal chemist, they will work on an enormous number of compounds but perhaps only have the privilege to be involved with a small number that reach the market, if any at all. But how should a chemist respond to the statement “That will never be a drug!”? One answer is by educating themselves on how to predict human dose and pharmacokinetics and comparing them to known drugs. There are many methods available to do these types of estimates, but broadly they fall into three categories which are as follows: (a) allometric scaling, (b) in vitro to in vivo correlation type methods, and (c) physiological based pharmacokinetic models (PBPK). Before calculating a human dose, however, the following parameters become useful, if not critical, to estimating human dose:

a) Estimated human clearance
b) Levels of target engagement needed (e.g., IC_50_, IC_75_, IC_90_, > IC_90_, etc.) specific to the target and drug potency in the most relevant biological assay in order to calculate free drug needed for C_trough_
c) Absorption and distribution (e.g., % *F* and *V*_d_)

### Estimating Human Clearance

For estimating human clearance, several models exist, and it is not the purpose of this article to provide an extensive review on the subject. If the reader is interested, they are referred to Yim et al. for a more comprehensive understanding of predicting human clearance.^186^ The purpose of this section is to provide the medicinal chemist with a few simple and practical methods to estimate important human PK properties and human dose. One such simple method to approximate human clearance is by allometric scaling from preclinical species. Equation 1 can be applied, and examples of how to use it (along with unit conversions) can be found in the Supporting Information (Supporting_Information_1_useful_PK_tools), since most medicinal chemists (including this author) are capable of using simple-to-moderate and sometimes complex ADME/DMPK formulas, but it is often helpful to have a real example to double check units, math transformations, etc.

1 $${CL}_{h}={CL}_{rat}\times {\left(\frac{W_{h}}{W_{rat}}\right)}^{0.75}$$

A typical coefficient of 0.75 is used in this example, but other coefficients are also used, and the reader can explore the different options that are available.^187^ Alternatively, a simple back of the envelope calculation can be used as CL_human/kg_ = 0.152 CL_rat/kg_.^188^ Allometric scaling methods can be useful when human drug exposure and disposition are well predicted by preclinical species. However, there may be cases where a certain species has extraordinarily high or low PK parameters that are not well correlated to human values using allometric scaling methods. In addition, some species may have significant sex differences in metabolism but may not be reflected in human clinical trials; thus, care must be taken in understanding both species and sex differences when using allometric scaling models.^189^

A complementary method to allometric scaling is to use in vitro parameters to predict in vivo outcomes. This is often referred to as in vitro to in vivo correlation (IVIVc). For example, a common practice is to use in vitro metabolism measures from human liver microsomes or hepatocytes and predict the corresponding in vivo clearance. There are many published methods to do this, but one method in particular is highlighted in this perspective.^190^ This method takes the in vitro clearance values as determined by human liver microsomes and corrects for both blood and microsome binding values. Equations 2 and 3 in this reference are valuable tools to both estimate human clearance but can also be used to compare IVIVc across preclinical species when correcting for *Q*, the liver blood flow of each preclinical species (e.g., rat in vitro clearance to rat in vivo clearance). These methods work when there is good correlation between in vitro microsomal clearance and in vivo clearnace, typically when major metabolic pathways are driven by oxidative enzymes found in in the liver. This method may not be the best for predicting in vivo clearance when there are major Phase 2 metabolic pathways, or extrahepatic clearance.

2 $${CL}_{blood}=\frac{Q\times f_{u\left(blood\right)}\times {CL}_{\mathrm{int}}^{\prime }}{Q+f_{u\left(blood\right)}\times {CL}_{\mathrm{int}}^{\prime }}$$

3 $${CL}_{blood}=\frac{Q\times f_{u\left(blood\right)}\times \frac{{CL}_{\mathrm{int}}^{\prime }}{f_{u\left(mic\right)}}}{Q+f_{u\left(blood\right)}\times \frac{{CL}_{\mathrm{int}}^{\prime }}{f_{u\left(mic\right)}}}$$

*f*_u_ = fraction unbound and CĹ_int_ = intrinsic clearance.

As mentioned above, an example of the formula as applied (along with unit conversions) can be found in Supporting Information (Supporting_Information_1_useful_PK_tools).

### Predicting Levels of Target Engagement Needed (e.g., IC_50_, IC_75_, IC_90_, > IC_90_, etc.)

Of equal importance to understanding the pharmacokinetic disposition of the compounds of interest, one must have a practical view of how much free drug is needed to elicit a pharmacological effect and how much of the target needs to be engaged to get the desired response. A recent article by Barrow and Lindsley stressed the importance of understanding the PK/PD relationships for drug discovery programs and provided important arguments on why it is critical to measure and understand.^191^ In early discovery, such as hit-to-lead, it may not be possible to estimate the levels of target engagement or PK/PD needed on novel targets, but nonetheless one can use generic estimates (e.g., IC_90_ coverage of free drug) until real data are generated on the program. For targets that are well-known, literature evidence or tool compounds may be available to provide this early assessment. For novel targets, extensive work may be required to establish a reliable preclinical model and a tool compound with good in vivo properties that will allow for this understanding. In the absence of a reliable model and tool, teams may choose to estimate with a range of free drug coverage such as IC_50_, IC_75_, IC_90_, and > IC_90_ in their dose prediction model until definitive data can be generated. This is an important parameter to understand since targets that require continuous coverage at higher target engagements (e.g., > IC_90_) may subsequently require either a very potent compound or a compound that can maintain very high levels of free drug with safe exposure levels or perhaps both. As a simple rule of thumb, IC_75_ ∼ 3× the IC_50_ and IC_90_ is ∼10× the IC_50_ and can be used to estimate back-of-the-envelope levels of free drugs that are needed. These rules apply for conventional reversible drugs that interact with the receptor and are not advised with covalent compounds or compounds that have a nontraditional kinetic profile (such as long residency time compounds where the IC_50_ does not reflect the real target potency).

### Predicting Absorption and Distribution

As is widely known, in vitro permeability models such as Caco-2 and MDCK (Madin–Darby canine kidney cells) are useful surrogates in understanding whether compounds have good passive permeability and/or efflux potential.^192^ However, these models cannot be used with confidence to quantitatively predict the value for oral bioavailability. For example, a molecule may have excellent oral absorption but may have a very rapid first pass metabolism that may subsequently limit the oral bioavailability. Early preclinical assessments in mice or rats are the most valuable predictors for this property. This is of course more expensive and more animal intensive than in vitro models; however, it is more accurate. In some cases, compounds can be run in a “cassette”, that is, multiple compounds dosed at the same time and analyzed independently. This can be a cost-effective way to gain an early understanding of oral bioavailability, but caveats must be noted such as the potential for DDI in the cassette that may alter the pharmacokinetics of other compounds in the mixture. There are models which are now public to predict oral bioavailability, such as recently reported by Zhang et al.^193^ As of the time of this writing, they have provided an open access tool to provide stratified probabilities of human oral bioavailability. As with all open access tools, care must be taken when sharing structures of proprietary compounds, and it would not be advisable to do so if the compounds were of proprietary importance and have intellectual property value. Nonetheless, the methods and rationale of such a technique are reported and available.

There are also published methods for allometric scaling for V_*d*_. An example reference can be found in the manuscript by Jones et al.^194^ Among these methods, a single species allometry model from rat to human may be of the most practical use for the medicinal chemist in early dose-to-human predictions (eq 4).

4 $$Human\;{VD}_{ss}=\left(rat\;{VD}_{ss}\right)\times \left(\frac{human\;f_{u}}{rat\;f_{u}}\right)$$

*f*_u_ = fraction of unbound plasma.

For more detailed predictions of volume of distribution, the reader is referred to other methods in the Jones paper,^194^ such as two species allometry methods.

Another parameter that may be of interest to extrapolate is the half-life (*t*_1/2_). The reader is pointed to several useful references in this regard.^195,196^ For half-life, a simple allometric scaling factor of ∼4.3 from rat to human can be used as an estimate in early stages of drug discovery as a practical guide for medicinal chemists.^196^ The caveats with using this approach are similar to the ones for allometric scaling of clearance in that such an approach assumes that the in vivo DMPK properties are predictable across species.

Pulling these concepts together, equations and tools exist to take these properties and predict a potential human dose. For medicinal chemists, simple one-compartment models are by far the easiest to model and may be used as a first estimation of human dose. However, these models may not predict compounds with complex PK and complex in vivo distribution. With that being noted, the reader is directed to the Supporting Information (Supporting_Information_1_useful_PK_tools) for more detailed equations and tools that may be used for predicting parameters for human dose prediction.

Some organizations adopt an early dose-to-human projection tool that can help monitor progress across a series. As an example, a team at Johnson and Johnson recently reported on a dose prediction tool implemented at the point of design. The strategy utilizes the ability to predict a variety of in vitro methods such as PPB, intrinsic clearance, MDCK permeability, and others. The model allows teams to estimate the oral dose required for a 10 nM trough exposure for 12h dosing interval.^197^ Within this paper, readers may also find some very useful formulas and derivatizations of the dose equation found in the text and also elaborated in the Supporting Information (Supporting_information_1_useful PK tools).^197^ Several other useful web-based tools are available as of the time of this writing, for example, the Medicinal Chemistry Toolkit provided by Molmatinif offers a dose to man tool,^198^ based on scaling equations from McGinnity et al.^199^ Of special utility within this tool and tools of this type is that they can be used to estimate the exposure relative to a minimum efficacious concentration (MEC). The MEC can be generated using inputs from target engagement assessments, as discussed above. Finally, another useful set of freely available tools that can be used to estimate human PK and dose projection is sponsored by Medicines for Malaria Venture.^200^ These tools are available at the time of this publication. Taken together, tools such as these are helpful to chemists in both the early phase and later phases of drug discovery as they can help shape an understanding of the estimated human dose and exposure projections, which can then be translated by the chemists into structural changes that can influence these parameters.

A final reflection on this set of approved drugs, although not quantifiable, is that there are several examples of uncommon or unexpected chemotypes. This category is of course highly subjective, and as the expression goes “one person’s trash is another person’s treasure”. In this authors’ experience, medicinal chemists do not often agree on what is or what is not drug-like. Many vigorous debates have been held among medicinal chemists on this topic over the years, and many will be held in the future. It is one of the great sports in medicinal chemistry. In the spirit of this Perspective and this view as a backdrop, Table 5 is a short attempt to list some of the less common chemotypes based on this authors experience. For example, acetylenes are a chemotype that potentially could metabolize to a ketene and result in mechanism-based inactivation of CYP enzymes.^201^ However, there are three examples in this report that have been approved in three different indications (in addition to other acetylenes that are on the market.) A styrene group is also the one that may be frowned upon by medicinal chemists, given the potential for intrinsic reactivity and potential oxidative liability. There are two examples of styrene moieties found in the list of approved drugs in this report (Table 5). There are also three examples found with N–O bonds, such as hydroxamic acids and oxime-like functionalities. Given the relative paucity of oxime and alkyl-oximes as drugs, it is safe to say that most chemists do not know their relative metabolic fate. There are also four examples of aromatic bromides. Although there is nothing intrinsically wrong with aromatic bromides, they are not common in approved drugs, perhaps due in part to the higher amount of lipophilicity that they bring relative to their chloro- or fluoro-phenyl counterparts. There are also several nitro functional groups found in this set of approved drugs (Table 5). However, it should be noted that at least two of these drugs are among the class of nitro-containing antibiotics and antiprotozoals, where the mechanism relies on the reduction of the nitro group to inhibit the growth of the pathogen. A final example of less common chemotypes in Table 13 is the *N*-oxide found in arimoclomol. These examples will hopefully continue to inspire chemists to think about what is possible and not get trapped into dogma and unsubstantiated views of what is and what is not drug-like. In many cases, our views have been shaped by an example or two of a functional group that has caused problems, but with a scientific understanding of the issue and the ability of medicinal chemists to manipulate the properties that regulate ADME, sometimes these issues can be mitigated in the context of a different chemical scaffold.

**Table 5:** Chemotypes in Table 1 That May Be Considered Less Common among Medicinal Chemists

chemotype	examples
Acetylene	Futibatinib (Table 1, Entry 56)
	Lenacapavir (Table 1, Entry 54)
	Crinecerfont (Table 1, Entry 89)
Styrene	Palovarotene (Table 1, Entry 61)
	Elafibranor (Table 1, Entry 100)
N–O bonds (e.g., hydroxamic acid or oximes)	Givinostat (Table 1, Entry 102)
	Arimoclomol (Table 1, Entry 96)
	Selumetinib (Table 1, Entry 8)
Aromatic bromide	Selumetinib (Table 1, Entry 8)
	Lonafarnib (Table 1, Entry 1)
	Ripretinib (Table 1, Entry 20)
	Aprocitentan (Table 1, Entry 91)
Nitro	Nifurtimox (Table 1, Entry 3), although this is the MOA
	Opicapone (Table 1, Entry 21), also an *N*-oxide and catechol
	Fexinidazole (Table 1, Entry 30)
*N*-oxide	Arimoclomol (Table 1, Entry 96)

## Conclusions

It is hoped that medicinal chemists may view this analysis as a reference point to help guide the design of potential future drugs. It is far too easy to layer a set of rules on what does not make a good drug. It is much harder to be the champion for a compound that others perceive as “un-drug like”. The data in this analysis illustrate that profiles of recently approved oral drugs appear to have broad ranges of PK properties, dose, dose frequencies, and safety warnings and are not easily defined by a strict dose and a set of ideal PK parameters. Care must be taken to avoid implementing overly prescriptive DC criteria that on the surface allow for early derisking of expensive and long future studies but may slow down progress of meaningful and innovative medicines that can reach patients in need. In addition to the analysis of properties, another metric also stands out and is worth noting, which is that of the 104 small-molecule drugs approved between 2020 and 2024, 42 out of 104 approvals are considered FIC. Furthermore, 59 out of 104 approvals have an ODD. This highlights the exceptional innovation and therapeutic breakthroughs that are having a direct impact on patients’ lives due to the medicinal chemists and drug discovery scientists that push forward new ideas, sometimes against strong headwinds. The industry will likely continue to have robust debates among scientists such as the “That will never be a drug!” vs “Why not?”, but it is hoped that with data in hand, and the ability to predict safe human doses, more drugs will be developed that treat patients in need.

## Supplementary Material





## Notes

Useful PK tools (PDF)
Data files (XLSX)

## Abbreviations

ACL: Adenosine triphosphate-citrate lyase
ADHD: Attention Deficit/Hyperactivity Disorder
ALGS: Alagille syndrome
ALK: anaplastic lymphoma kinase
ALS: amyotrophic lateral sclerosis
ANCA: antineutrophil cytoplasmic autoantibody
BID: bis in die (twice per day)
C5a1: BTK, Bruton Tyrosine Kinase, Complement Component 5a Receptor 1
CDKL5: cyclin-dependent kinase-like 5 (CDKL5) deficiency
CGRP: calcitonin gene-related peptide
CFTR: Cystic Fibrosis Transmembrane Conductance Regulator
COMT: Catechol-O-methyl transferase; DC criteria, Development Candidate criteria
CMV: Cytomegalovirus
DDI: drug–drug interactions
DILI: drug induced liver injury
DMD: Duchenne muscular dystrophy
EGFR: Epidermal Growth Factor Receptor
FGFR2: Fibroblast Growth Factor Receptor 2
FIC: first-in-class
FLT3: Fms-like Tyrosine Kinase 3
GABA: γ-aminobutyric acid
GLP: Good Laboratory Practice
GPA: granulomatosis with polyangiitis
GnRH: Gonadotropin-Releasing Hormone
HER2: Human Epidermal Growth Factor Receptor 2
HIF-PH: Hypoxia-inducible factor prolyl hydroxylase
HIV-1: human immunodeficiency virus type 1
HPGS: Hutchinson-Gilford Progeria Syndrome
HV: Healthy Volunteers
IDH-1: isocitrate dehydrogenase-1
Iga: Immunoglobulin A
IND: Investigational New Drug
JAK: Janus Kinase
MAPK: mitogen activated protein kinase
MBC: Metastatic Breast Cancer
MDCK: Madin–Darby canine kidney cells
NK3: Neurokinin 3 Receptor
Nrf2: Nuclear factor (erythroid-derived 2)-like 2
NSCLC: Nonsmall Lung Cell Carcinoma
PB: Phenylbutyrate
PDGFRα: Platelet-Derived Growth Factor Receptor Alpha
PDPL: Processing-Deficient Progeroid Laminopathy
PFIC: Progressive familial intrahepatic cholestasis
PK: pharmacokinetic
PI3Kδ: phosphoinositide 3-kinase delta
sGC: Soluble Guanylate Cyclase
PPAR: Peroxisome Proliferator-Activated Receptor
ROCK2: Rho-associated Protein Kinase 2
SMN2: Survival motor neuron 2
SGLT2: sodium-glucose cotransport protein 2
TID: ter in die (three times daily)
QD: quaque die (once daily)
TUDCA: taurursodiol
TPP: Therapeutic Product Profile
VEGF: Vascular Endothelial Growth Factor
VHL: Von Hippel-Lindau
WHIM: Warts, Hypogammaglobulinemia, Infections, and Myelokathexis
