# Supplementary material for: What Do Oral Drugs Really Look Like? Dose Regimen, Pharmacokinetics, and Safety of Recently Approved Small-Molecule Oral Drugs
Source: J Med Chem. 2025 Nov 18;68(22):23751–80. doi: 10.1021/acs.jmedchem.5c02863 (PMC12670434; doi:10.1021/acs.jmedchem.5c02863)
Supplement: Supplementary file 1 [file jm5c02863_si_001.pdf]

What do oral drugs really look like? Dose regimen, pharmacokinetics and safety of recently approved small molecule oral drugs

Dean G. Brown

Jnana Therapeutics, One Design Center Pl Suite 19-400, Boston, MA 02210

SUPPORTING INFORMATION

Table of Contents **Page**

Table of Contents	Page
Example of allometric scaling for rat	2
Allometric scaling of VDss	2
Simple allometric scaling from rat to human for $t_{1/2}$	2
Example of in vitro scaling of human blood clearance	3
Calculation of dose, based on a projected required minimal concentration (C)	5
Other useful tools and calculations	5
References	6

1) Example of allometric scaling for rat where CL = 19 mL/min/kg:

$$CL_h = CL_{rat}\ x \left(\frac{W_h}{W_{rat}}\right)^{0.75} \quad (1)$$

$$CL_h = CL_{rat}\ x \left(\frac{70\ kg}{0.25\ kg}\right)^{0.75}$$

$$CL_{rat} = 19\ \text{mL/min/kg} = 0.285\ \text{L/h}$$

$$CL_h = 0.285\ L/h\ x \left(\frac{70\ kg}{0.25\ kg}\right)^{0.75}$$

$$CL_h = 0.285\ L/h\ x\ (280)^{0.75}$$

$$CL_h = 19.5\ L/h$$

$$CL_h = 19.5\frac{L}{h} = 4.64\frac{mL}{min}/kg$$

For body weight guides see the following reference by Nair et al.[1]

2) Allometric scaling of VDss:

There are many published methods of allometric scaling for VDss. An example reference can be found in the reference by Jones et al.[2] Amongst these methods, a single species allometry model from rat to human may be of the most practical use for the medicinal chemist in early dose-to-human predictions.

$$Human\ VDss = (Rat\ VDss)x\left(\frac{Human\ fu}{Rat\ fu}\right) \quad (2)$$

f_u = fraction unbound plasma

For more detailed predictions of VDss, methods in the Jones paper,[2] such as two species allometry, may be used instead.

3) For half-life, a simple allometric scaling factor of ~4.3 from rat to human can be used as an estimate early stages of drug discovery as a practical guide for medicinal chemists.[3,4] The

caveats with using this approach are similar to the ones for clearance, in that such an approach assumes that the in vivo DMPK properties are predictable across species.

4) Example of in vitro scaling of human blood clearance from in vitro properties (human microsomal clearance) using equations (3) and (4)

$$CL_{blood} = \frac{Q \times f_{u(blood)} \times CL'_{int}}{Q + f_{u(blood)} \times CL'_{int}} \quad (3)$$

$$CL_{blood} = \frac{Q \times f_{u(blood)} \times \frac{CL'_{int}}{f_{u(mic)}}}{Q + f_{u(blood)} \times \frac{CL'_{int}}{f_{u(mic)}}} \quad (4)$$

CL'int is a scaled property that can be converted from the in vitro half-life using the following equation. For most purposes, this scaled property is reported from individual labs and calculated from the intrinsic clearance and will not need to be converted by medicinal chemists.

$$CL'_{int} = \frac{0.693}{\text{in vitro } T_{1/2}} \times \frac{\text{mL incubation}}{\text{mg microsomes}} \times \frac{\text{45 mg microsomes}}{\text{gm liver}} \times \frac{\text{20 gm liver}}{\text{kg BW}} \quad (5)$$

Examples from equation 3: Assume in vitro CL'_{int} = 8 mL/min/kg, f_u = 0.12, Q = 21 mL/min/kg and blood-to-plasma ratio equals 0.78. Correct for $f_{u(blood)}$ by taking fraction unbound in plasma (f_u) and correcting for blood-to-plasma ratio:

Example: f_u = 0.12, and blood-to-plasma-ratio is 0.78, then $f_{u(blood)}$ = 0.15

$$CL_{blood} = \frac{\frac{21\,mL}{min}/kg \times 0.15 \times 8\,\frac{mL}{min}/kg}{\frac{21\,\frac{ml}{min}}{kg} + 0.15 \times 8\,\frac{mL}{min}/kg} \quad (3)$$

$$CL_{blood} = 1.13\,\frac{mL}{min}/kg = \frac{25.2\,\frac{mL}{min}/kg}{22.2\,mL/min/kg}$$

Examples from equation 4: Assume in vitro CL'_{int} = 8 mL/min/kg), f_u = 0.12 and Q = 21 mL/min/kg, and fu(mic) = 0.11

$$CL_{blood} = \frac{\frac{21\,\frac{mL}{min}}{kg} \times 0.15 \times \frac{8\,\frac{mL}{min}/kg}{0.11}}{\frac{21\,\frac{mL}{min}}{kg} + 0.15 \times \frac{8\,\frac{mL}{min}/kg}{0.11}} \quad (3)$$

$$CL_{blood} = 7.17 = \frac{229\,\frac{mL}{min}/kg}{31.9\,\frac{mL}{min}/kg}$$

For a table of liver blood flows, see DrugHunter cheatsheet[5]

5) Calculation of dose, based on a projected required minimal concentration (C). A recent example of this equation is shown, and also for using predictive properties early in a drug discovery application.[6]

$$Dose = \frac{BW\ x\ MW\ x\ C\ x\ (K_a - K_e)\ x\ VD_{ss}}{AR\ x\ f_u\ x\ 1000\ x\ F\ x\ K_a\ x\ (e^{-k_e\tau} - e^{-k_a\tau})}$$

C = target concentration (µM)

VD_{ss} = volume of distribution at steady state (in L/kg). Can be extrapolated from in vivo experiments using single species allometry models (see examples above)

Cl is the clearance (in L/kg/h)

AR is the accumulation ratio (unitless), assume 1 if not known

f_u = the fraction unbound

F = oral bioavailability as a fraction (e.g. 0.7, not 70%)

k_a = the absorption constant (h^{-1}),

k_e = elimination constant (h^{-1})

BW is the bodyweight (kg)

τ = the dosing interval (in h, e.g. 12h, 24 h)

6) Other useful tools and calculations:

- ✓ Conversion from ng/mL to µM : Simply divide ng/mL by the MW (g/mol).
  - Example 1800 ng/mL for a 400 MW cpd = 4.5 µM
  - Conversion from uM to ng/mL : Multiply uM concentration x MW
  - Example 4.5 uM concentration of a 400 MW cpd = 1800 ng/mL
- ✓ Calculate ICanything from IC_{50}: https://www.graphpad.com/quickcalcs/ecanything1/
  - A simple rule of thumb IC_{75} = ~3x IC_{50}. IC_{90} ~9x IC_{50}
- ✓ How to calculate the free drug at a given ICxx:

- Assume you need to achieve IC_{90} coverage of a 20 nM compound at $C_{trough,\ unbound}$, which is 95% plasma protein bound.
- IC_{90} = 180 nM. Total drug would be 180nM /0.05 free = 3600 nM.

✓ Useful freely available dose to human calculators

- Medicinal Chemistry Toolkit, by Molmatinif[7]
- Medicines for Malaria Venture tools[8]
- Useful equations (University of Florida[9]
- DrugHunter PK cheatsheat[5]

References:

(1) Nair, A. B.; Jacob, S. A Simple Practice Guide for Dose Conversion between Animals and Human. *J. Basic Clin. Pharm.* **2016**, *7* (2), 27–31. https://doi.org/10.4103/0976-0105.177703.

(2) Jones, R. D.; Jones, H. M.; Rowland, M.; Gibson, C. R.; Yates, J. W. T.; Chien, J. Y.; Ring, B. J.; Adkison, K. K.; Ku, M. S.; He, H.; Vuppugalla, R.; Marathe, P.; Fischer, V.; Dutta, S.; Sinha, V. K.; Björnsson, T.; Lavé, T.; Poulin, P. PhRMA CPCDC Initiative on Predictive Models of Human Pharmacokinetics, Part 2: Comparative Assessment of Prediction Methods of Human Volume of Distribution. *J. Pharm. Sci.* **2011**, *100* (10), 4074–4089. https://doi.org/10.1002/jps.22553.

(3) Caldwell, G. W.; Masucci, J. A.; Yan, Z.; Hageman, W. Allometric Scaling of Pharmacokinetic Parameters in Drug Discovery: Can Human CL, Vss and T1/2 Be Predicted from in-Vivo Rat Data? *Eur. J. Drug Metab. Pharmacokinet.* **2004**, *29* (2), 133–143. https://doi.org/10.1007/BF03190588.

(4) Gunaydin, H.; Altman, M. D.; Ellis, J. M.; Fuller, P.; Johnson, S. A.; Lahue, B.; Lapointe, B. Strategy for Extending Half-Life in Drug Design and Its Significance. *ACS Med. Chem. Lett.* **2018**, *9* (6), 528–533. https://doi.org/10.1021/acsmedchemlett.8b00018.

(5) *PK Cheat Sheet*. Drug Hunter. https://drughunter.com/resource/pk-cheat-sheet (accessed 2025-10-03).

(6) Van Rompaey, D.; Ray Chaudhuri, S.; Ahmad, M.; Cisar, J.; Van Den Bergh, A.; Ash, J.; Wu, Z.; Bryan, M. C.; Edwards, J. P.; DesJarlais, R.; Wegner, J. K.; Ceulemans, H.; Mitra, K.; Polidori, D. Toward Dose Prediction at Point of Design. *J. Med. Chem.* **2024**, *67* (24), 22282–22290. https://doi.org/10.1021/acs.jmedchem.4c02385.

(7) *Medicinal Chemistry Toolkit 2*. https://molmatinf.com/medchemtoolkit/ (accessed 2025-10-03).

(8) *Computational chemistry guides & tools*. Medicines for Malaria Venture. https://www.mmv.org/research-development/computationalchemistry (accessed 2025-10-03).

(9) Useful Pharmacokinetic Equations, UF College of Pharmacy. https://pharmacy.ufl.edu/files/2013/01/5127-28-equations.pdf (accessed 2025-10-03).
